# *Iris sanguinea* is conspecific with *I. sibirica* (Iridaceae) according to morphology and plastid DNA sequence data

**DOI:** 10.7717/peerj.10088

**Published:** 2020-10-01

**Authors:** Eugeny Boltenkov, Elena Artyukova, Marina Kozyrenko, Andrey Erst, Anna Trias-Blasi

**Affiliations:** 1 Botanical Garden-Institute, Far Eastern Branch, Russian Academy of Sciences, Vladivostok, Russia; 2 Federal Scientific Center of the East Asia Terrestrial Biodiversity, Far Eastern Branch, Russian Academy of Sciences, Vladivostok, Russia; 3 Central Siberian Botanical Garden, Siberian Branch, Russian Academy of Sciences, Novosibirsk, Russia; 4 Tomsk State University, Tomsk, Russia; 5 Royal Botanic Gardens, Kew, Richmond, UK

**Keywords:** Chloroplast DNA, *Iris* subser. *Sibiricae*, Molecular phylogeny, Morphology, Nomenclature, Taxonomy

## Abstract

A taxonomic revision of *Iris* subser. *Sibiricae* is provided based on morphological and molecular analyses and the study of protologues and original material. Two to three species have been recognized in this subseries by botanists. To address the question of species delimitations and relationships within this group, we analyzed four non-coding regions of plastid DNA (*trn*S–*trn*G, *trn*L*–trn*F, *rps*4*–trn*S*^GGA^*, and *psb*A–*trn*H) for samples from 26 localities across the distribution ranges of two currently recognized species, *I. sanguinea* and *I. sibirica*. Variance analysis, based on nine characters, revealed no separation between taxa. Moreover, no morphological character could be used to define clear boundaries between taxa. Our results strongly support that *I.* subser. *Sibiricae* is monotypic and comprises only *I. sibirica*, instead of two or three species.* Iris sibirica* is morphologically variable and one of the most widespread Eurasian species of Iridaceae. Previously accepted taxa,* I. sanguinea* and *I*. *typhifolia*, are synonymised with *I. sibirica* and also two names, *I. orientalis* and* I. sibirica* var. *haematophylla*, which are typified here, are placed in the synonymy of *I. sibirica*. Information on the distribution of *I. sibirica* and the main features used to distinguish between *I. sibirica* and *I*. subser. *Chrysographes* species are provided.

## Introduction

*Iris* L. is the largest, most widespread in Iridaceae distributed mainly in the temperate zones of the Northern Hemisphere. *Iris* is a taxonomically difficult genus. Its generic limits are controversial, and recent data seem to favour a much narrower circumscription (*Crespo, Martínez-Azorín & Mavrodiev, 2015*). However, the infrageneric composition and circumscription of *Iris* is questionable (*Boltenkov et al., 2018*). Therefore, we believe that additional studies are needed, and thus, a conservative taxonomy is here applied (*Mathew, 1989; Wilson, 2009*).

While revising *I*. sect. *Limniris* Tausch, we find that the taxonomy of *I.* ser. *Sibiricae* (Diels) G.H.M.Lawr. still remains unclear. Plants of this Eurasian group are rhizomatous herbs distinguished from all the other *Iris* species, except *I. clarkei* Baker ex Hook.f., by having a hollow flowering stem. The infrageneric taxon *Sibiricae* was first described by Diels (1930) as a subsection, including eight species with a short tube, a triangular elongated stigma, narrow grassy leaves, in cross-section triangular capsules, and disc-shaped or nearly cubical seeds. These species were later subdivided into two groups on account of their chromosome numbers (*Simonet, 1934*), morphology and geographical distribution (Grey-Wilson, 1971; Lenz, 1976). The autonymic subseries of *I*. ser. *Sibiricae* includes well-known garden ornamentals, with 2*n* = 28 chromosomes (*Löve, 1975; Probatova, 2006*), that hybridise easily both in the garden and in the wild (*McEwen & McGarvey, 1978; Grey-Wilson, 2012*), and are known to horticulturists under the name of Siberian irises. The other group, *I.* subser. *Chrysographes* (Simonet) L.W.Lenz, comprises species with 2*n* = 40 chromosomes, and are known to horticulturists as Sino-Siberian irises (*Waddick & Zhao, 1992*). These latter irises are native to southwestern China (mainly Yunnan and Sichuan provinces) and eastern Himalayas, and occur at high elevations (*Zhao, Noltie & Mathew, 2000; Grey-Wilson, 2012*). The distinctness of these two groups within *I.* ser. *Sibiricae* was also supported by previous molecular studies (*Tillie, Chase & Hall, 2000; Wheeler & Wilson, 2014; Crespo, Martínez-Azorín & Mavrodiev, 2015*).

The species’ circumscription of Siberian irises differed among later botanists, who distinguished either two (*McEwen & McGarvey, 1978; Mathew, 1989; Doronkin, 2012*) or three species (*Rodionenko, 2007; Zhao, Noltie & Mathew, 2000; Grey-Wilson, 2012; Crespo, Martínez-Azorín & Mavrodiev, 2015*) in this group: *I. sanguinea* Hornem., *I. sibirica* L., and *I. typhifolia* Kitag.

*Iris sibirica* was first described by Linnaeus (1753) from Austria, Switzerland, and Siberia. Authors from the end of the 19th century (e.g.,  Baker, 1877; Hooker, 1899) believed that *I. sibirica* is one of the most widespread species of Iridaceae in Eurasia, extending from Central Europe to Japan. Therefore, *I. sibirica* has been considered as a single species including several varieties (*Regel, 1867; Baker, 1877; Maximowicz, 1880; Komarov, 1901; Dykes, 1910*).

*Iris sanguinea* was formally described by Hornemann (1813) based on cultivated plants from the Botanical Garden of Copenhagen, Denmark. Subsequently, *I. sanguinea* was reduced to a variety of *I. sibirica*, i.e., *I. sibirica* var. *sanguinea* (Hornem.) Ker Gawl., characterized by having young leaves often red-tinged at base. Some authors (e.g., *Spach, 1846; Ledebour, 1852*) cited this variety under the name *I. sibirica* var. *haematophylla* Besser. At the same time, plants from the eastern regions of Eurasia were indicated under the names *I. sibirica* var. *sanguinea*, *I. sibirica* var. *haematophylla*, and *I. sibirica* var. *orientalis* (Schrank) Baker. Koidzumi (1926) re-established *I. sanguinea*, indicating a distribution range including Japan, Dauria (currently Transbaikal region), and the Amur River basin. As a result, this taxon was accepted as being native to temperate regions of East Asia by all subsequent authors (e.g., *Pavlova, 1987; Mathew, 1989*), or it was cited under the illegitimate name *I. orientalis* Thunb. (e.g., *Dykes, 1912; Diels, 1930; Fedtschenko, 1935; Lawrence, 1953*).

It has been stated that *I. sanguinea* and *I. sibirica* are morphologically barely distinguishable (*Komarov, 1901; Dykes, 1912; Grubov, 1977*), and their identification is mostly based on the inflorescence structure (*McEwen & McGarvey, 1978; Mathew, 1989; Grey-Wilson, 2012*). In the *I*. subser. *Sibiricae* species, the inflorescence is cymose and formed by the terminal head of flowers and one or two lateral heads (Szöllösi et al., 2011; Skrypec & Odintsova, 2017). According to several authors (*Dykes, 1912; Mathew, 1989; McEwen & McGarvey, 1978; Grey-Wilson, 2012*), the typical *I. sanguinea* individuals generally produce stem bearing the terminal head, while *I. sibirica* individuals produce a stem with terminal and lateral heads. According to Skrypec & Odintsova (2017), *I. sibirica* inflorescences have a high morphological variability in the number of flowers, their position, and the flowering order. Other studies (*Dénes, Juhász & Salamon-Albert, 2008; Szöllösi et al., 2011*) indicated that the inflorescence features in *I. sibirica* vary through years and depend on climatic parameters.

*Iris typhifolia*, the third species recognized in *I.* subser. *Sibiricae* by some authors, was described by Kitagawa (1934) as a Chinese endemic on the basis of one specimen. This specimen was collected in the northern part of the Beiling District (currently Shenyang City, Liaoning Province) and originally identified as *I. sibirica* (see Taxonomic treatment below). Kitagawa (1934) specified that *I. typhifolia* is distinct from other irises by having slender twisted leaves. Waddick & Zhao (1992) suggested that *I. typhifolia* differs from *I. sanguinea* by its narrow leaves, generally about 0.2 cm wide. Nevertheless, Grey-Wilson (2012) noticed that the cultivated plants of *I. typhifolia* appeared to differ from the original description (0.15–0.22 cm wide) in having broader leaves.

Fedtschenko (1949) noticed that the eastern boundary of the distribution range of *I. sibirica* is the *Sayan* Mountains in southern Siberia (Russia). According to recent studies (McEwen & McGarvey, 1978; Mathew, 1989; Galanin, 2009; Grey-Wilson, 2012), the identification of *I. sanguinea* and *I. sibirica* has often been based on their geographical origin: *I. sibirica* has been considered to be distributed in Europe and Western Siberia, while *I. sanguinea* has been considered to occur in East Asia, eastward Lake Baikal (also see Global Biodiversity Information Facility, 2020). *Iris typhifolia* has been reported from the same Chinese provinces where *I. sanguinea* has also been reported (Zhao, Noltie & Mathew, 2000). Furthermore, it has recently been found that the typical plants of *I. typhifolia* described by *Kitagawa (1934)* are not found in the type locality, or in any other area in Liaoning Province whereas plants matching *I. sanguinea* have been recorded in this province (*Zheng et al., 2017*).

Integrative approaches combining morphological and molecular data obtained from plastid DNA (cpDNA) and nuclear ribosomal DNA (nrDNA) are widely used to distinguish taxa at different taxonomic ranks (*Liu et al., 2012; Hu et al., 2015; Vicente, Alonso & Crespo, 2019*). The nrDNA spacer regions provide information useful for phylogenetic reconstructions in plant systematics, though intraindividual nrDNA polymorphism can lead to erroneous or ambiguous results (*Poczai & Hyvönen, 2010; Wilson, Padiernos & Sapir, 2016*). Numerous studies have highlighted the great value of applying chloroplast DNA (cpDNA) sequence data for species delimitation in *Iris* (Tillie, Chase & Hall, 2000; Wilson, 2004; Wilson, 2009; Wilson, 2011; Wilson, 2017; Guo & Wilson, 2013). In previous studies, we investigated the taxonomy of *I.* sect. *Psammiris* (Spach) J.J.Taylor (*Kozyrenko, Artyukova & Zhuravlev, 2009*) and *I*. ser. *Lacteae* Doronkin (*Boltenkov, Artyukova & Kozyrenko, 2016; Boltenkov et al., 2018*) based on cpDNA analysis (*Boltenkov, Artyukova & Kozyrenko, 2016; Boltenkov, 2018*).

To reconstruct the relationships among species within *I*. subser. *Sibiricae*, we used morphological and molecular data. Our aims are: (1) to compare the morphological characters of living plants and herbarium specimens from the distribution range of *I*. ser. *Sibiricae*; (2) to resolve the phylogenetic relationships of the *I*. subser. *Sibiricae* species and of some other series of *I*. sect. *Limniris* using four cpDNA regions; (3) to ascertain whether the genetic relationships among *I. sanguinea* and *I. sibirica* are consistent with their current taxonomic classification as separate species; and (4) to compare the results of morphological and molecular studies in order to evaluate the number of species in *I.* subser. *Sibiricae*.

## Materials & Methods

### Morphological study

**Figure 1:**
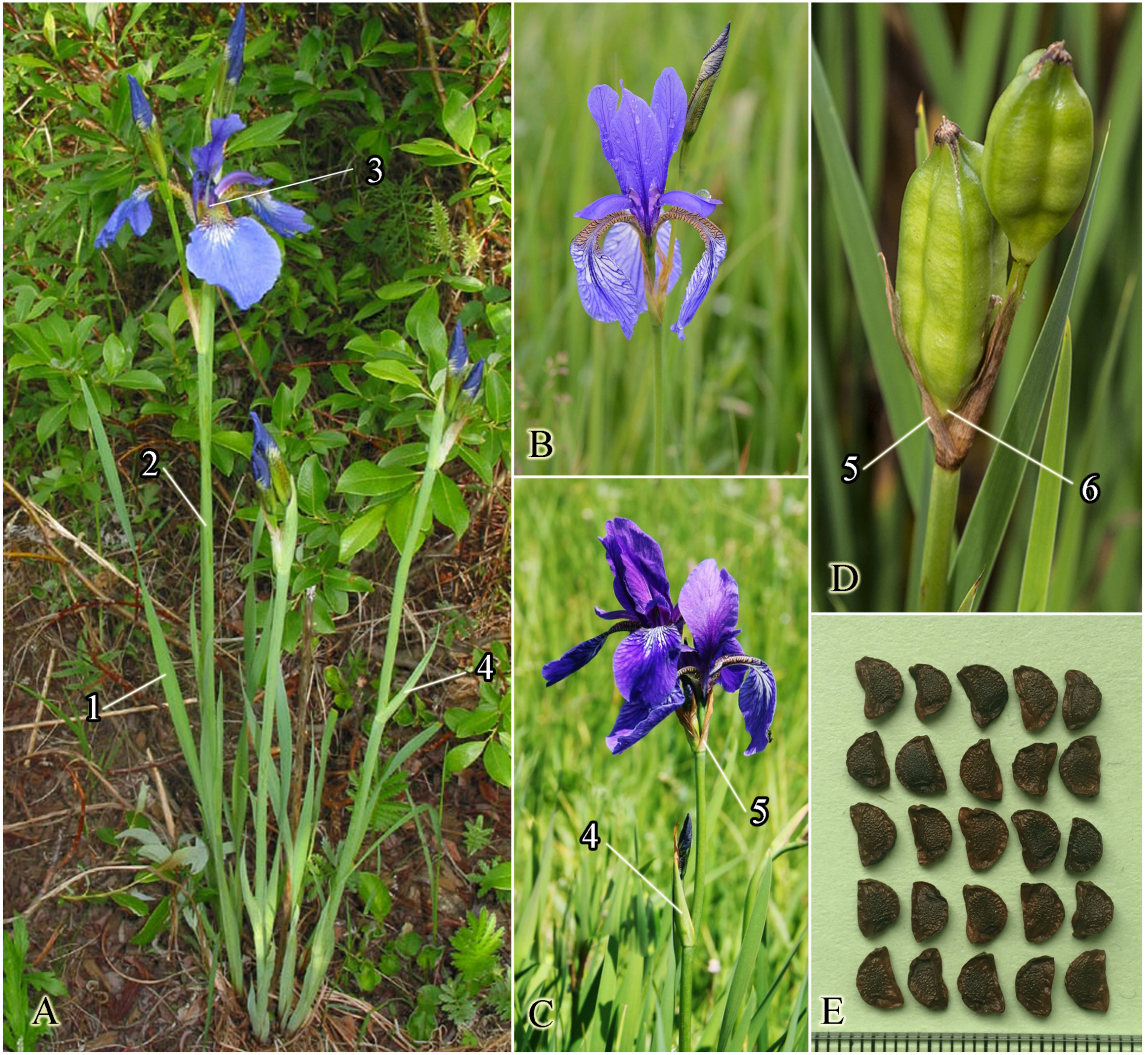
Photos of living plants of *Iris sibirica*. (A) Plant in habitat. (B) Inflorescence with the terminal head of two flowers. (C) Inflorescence with the terminal head and one lateral head. (D) Fruits. (E) Seeds.

The *I*. subser. *Sibiricae* species descriptions available in literature (*Krylov, 1929; Sergievskaya, 1972; Doronkin, 1987; Mathew, 1989; Pavlova, 1987; Zhao, Noltie & Mathew, 2000; Grey-Wilson, 2012*) were examined. We evaluated the thirteen characters, which were selected from those typically used in the literature together with those considered relevant according to our personal observations (see Fig. 1). These characters are listed in detail in Table 1. The original material of *I. sanguinea*, *I. sibirica*, *I. sibirica* var. *haematophylla*, *I. orientalis* Thunb., and *I*. *typhifolia* (see Taxonomic treatment below) was studied. In total, 224 scaled specimens of well-developed plants in flowering or fruiting were measured (see Appendix S1). The specimens of *I. sanguinea* and *I. sibirica* have been checked through high resolution images available in virtual herbaria (herbarium codes according to *Thiers, 2020*): ABGI and VBGI (https://botsad.ru/herbarium/), E (https://data.rbge.org.uk/search/herbarium/), MHA and MW (https://plant.depo.msu.ru/), NS and NSK (http://herb.csbg.nsc.ru:8081/#fuzzy-label), PI, PRC and WU (https://herbarium.univie.ac.at/database/search.php). For *I*. *typhifolia*, 48 specimens were used: 27 specimens from the Chinese botanist Yu-Tang Zhao, an expert on Chinese Iridaceae (e.g., *Waddick & Zhao, 1992; Zhao, Noltie & Mathew, 2000*), collection at NENU, and also 21 specimens have been checked through images available in virtual Chinese herbaria ( http://www.cvh.ac.cn/). The morphological characters were measured using AxioVision 4.8 (Carl Zeiss, Germany), a freeware comprehensive images viewer.

**Table 1:** Morphological characters analysed in the *Iris* subser. *Sibiricae* species.

**No.**	**Character**	**Code**	**Remarks**
1	Rosette leaf length (cm)	LL*	Measured from base to apex for the longest leaf in rosette
2	Rosette leaf width (cm)	LW*	Measured in its broadest place for the broadest leaf in rosette
3	Flowering stem height (cm)	SH*	Measured from base of stem to base of bracts
4	Inflorescence structure	IS*	Classified as inflorescence with terminal head (1) or with terminal and one lateral head (2)
5	Number of flowers	NF*	Flowers per stem
6	Number of cauline leaves	NC*	Leaves arising on the flowering stem
7	Cauline leaf length (cm)	CL*	Measured from base to apex for the upper leaf
8	Bract length (cm)	BL*	Measured from base to apex for the outer bract
9	Pedicel length (cm)	PL*	Measured for the first blooming flower in the terminal head
10	Flower colour	FC	According to literature data
11	Fruit length (cm)	FL	Obtained for all fruits from the specimens at fruiting
12	Fruit shape	FS	Obtained from the specimens at fruiting
13	Seed shape	SS	According to literature data

**Note.**

Asterisk (*) indicates characters used in the variance analysis.

For morphometric data analysis, nine characters were used (see Table 1). In this study both parametric and non-parametric versions of a one-way variance analysis (ANOVA) were applied. The differences were considered significant at *p*-value <  0.05. As multiple statistical testing was performed, the calculated *p*-value was adjusted using the procedure proposed by Benjamini & Hochberg (1995). To test basic ANOVA assumptions Shapiro–Wilk test for normality and Levene’s test for equality of variances were used. The missing values in the original data table were imputed using corresponding median values according (*Kuhn & Johnson, 2018*). The Kruskal–Wallis test was chosen as a non-parametric ANOVA algorithm (*Dodge, 2008*). Principal components analysis (PCA) was used to visualize the distribution of the analyzed individuals over the space of morphometric characters. It was applied to all quantitative characteristics. Directions of principal components were described in the factor space by their highest correlation values (denoted by r) with original axes. Computations were performed by means of SciPy (*Virtanen et al., 2020*) and Scikit-Learn (Pedregosa et al., 2011) packages.

### DNA extraction, amplification and sequencing

**Figure 2:**
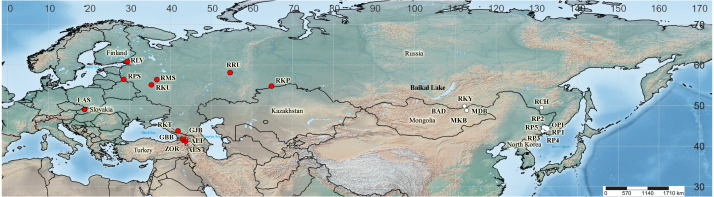
Map showing the geographical origin of *Iris* subser. *Sibiricae* samples analyzed in the present study (created with https://www.simplemappr.net, CC 1.0). Locality codes as in Table 2; cultivated plants (Sc1 and Sc2) are not mapped. Red circles –populations in the *I. sibirica* distribution range; white circles –populations in the *I. sanguinea* distribution range; black square –the locality of *I. sanguinea* from the Republic of Korea (Lee et al., 2017).

Sequences of four cpDNA regions were obtained for 44 specimens taken from wild populations, herbarium material and living collections. Among those, there were 20 from 13 localities in the *I. sibirica* distribution range, 22 from 11 localities in the *I. sanguinea* distribution range, and two plants were of unknown origin (Fig. 2). It was not possible to obtain samples from Japan and northeastern China, including Liaoning Province, where *I. typhifolia* was described from. Nevertheless, while searching GenBank for any sequences of four studied cpDNA regions of the *I*. subser. *Sibiricae* species, we found sequences of only either *psb*A*–trn*H or *trn*L*–trn*F for several accessions of *I. sibirica* and *I. typhifolia*, as well as *I. sanguinea* from Japan, northeastern China and the Republic of Korea (see Table S1). The sequences of four cpDNA regions from the complete chloroplast genome of *I. sanguinea* from the Republic of Korea (*Lee et al., 2017*) were included in the study. Our sampling also comprises representatives of three other series of *I*. sect. *Limniris*: (1) *I. laevigata* Fisch., *I. ensata* Thunb., and *I. pseudacorus* L. from *I*. ser. *Laevigatae* (Diels) G.H.M.Lawr.; (2) *I. lactea* Pall., *I. oxypetala* Bunge, and *I. tibetica* from *I*. ser. *Lacteae*; (3) *I. uniflora* Pall. ex Link from *I*. ser. *Ruthenicae* (Diels) G.H.M.Lawr. *Iris dichotoma* Pall. from *I.* subgen. *Pardanthopsis* (Hance) Baker was used as outgroup. The complete specimen list, including the sampling localities and the voucher information is given in Table 2.

**Table 2:** Sample information of the accessions used in the study.

**Code (N)**	**H**	**Locality, voucher**	**GenBank accession numbers*trn*H–*psb*A/*rps*4–*trn*S/*trn*S–*trn*G/*trn*L–*trn*F**
*I.* ser. *Sibiricae* subser. *Sibiricae*			
BAD (1)	H1	Mongolia, Badgir, *Dolgaleva s.n.* (VBGI*)	*LT627899/ LT628015/ LT628026/ LT628005*
MDB (1)	H1	Mongolia, Dornod, Bayan-Uul, *Gubanov 550* (MW)	LT978556/ LT981298/ LT984448/ LT984480
MKB (1)	H1	Mongolia, Khentii, Binder Somon, *Galanin s.n.* (VBGI)	LT978557/ LT981299/ LT984449/ LT984481
ORL (3)	H2	Russia, Primorsky Krai, Orlovka, *Boltenkov s.n.* (VBGI)	*LT627900/ LT628016/ LT628027/ LT628006*
RCH (5)	H2	Russia, Amur Oblast, Chingan State Nature Reserve, *Kudrin s.n.* (ARKH)	LT978531/ LT981273/ LT984423/ LT984456
RP1 (1)	H1	Russia, Primorsky Krai, Solovei Kluch, *Boltenkov s.n.* (VBGI)	LT978535/ LT981277/ LT984427/ LT984460
RP2 (1)	H2	Russia, Primorsky Krai, Khankaysky District, Il’inka, *Pshennikova s.n.* (VBGI)	LT978530/ LT981272/ LT984422/ LT984455
RP3 (3)	H3	Russia, Primorsky Krai, vicinity of Vladivostok, *Kuritskaya s.n.* (VBGI)	LT978534/ LT981276/ LT984426/ LT984459
RP4 (1)	H3	Russia, Primorsky Krai, Romanovka, *Chubar s.n.* (VBGI)	LT978533/ LT981275/ LT984425/ LT984458
RP5 (2)	H3	Russia, Primorsky Krai, Pokrovka, *Denisova & Talovskaya s.n.* (VBGI)	LT978532/ LT981274/ LT984424/ LT984457
RKP (1)	H4	Russia, Kurgan Oblast, Pritobolny District, *Fedotova s.n.* (NSK)	LT978536/ LT981278/ LT984428/ LT984461
RKT (1)	H4	Russia, Karachay-Cherkess Republic, Teberda, *Shilnikov s.n.* (cult.)	LT978529/ LT981271/ LT984421/ LT984454
RKU (1)	H5	Russia, Kaluga Oblast, Ugra National Park, *Reshetnikova et al. s.n.* (MHA)	LT978539/ LT981281/ LT984431/ LT984464
RKY (3)	H1	Russia, Zabaykalsky Krai, Mountain Steppe State Reserve, *Roenko s.n.* (VBGI)	LT978542/ LT981284/ LT984434/ LT984467
RLV (1)	H6	Russia, Leningrad Oblast, vicinity of Vyborg, *Boltenkov s.n.* (cult.)	LT978545/ LT981287/ LT984437/ LT984470
RMS (1)	H7	Russia, Moscow, Setun River valley, *Nasimovitch & Shchukin s.n.* (MHA)	LT978541/ LT981283/ LT984433/ LT984466
RPS (8)	H5	Russia, Pskov Oblast, Sebezhsky District, *Konechnaya s.n.* (LE)	LT978538/ LT981280/ LT984430/ LT984463
RRU (1)	H4	Russia, Udmurt Republic, Perevoznoye, *Melnikov s.n.* (LE)	LT978537/ LT981279/ LT984429/ LT984462
ALS (1)	H4	Armenia, Lori Province, Saratovka, *Khanjyan & Tumanyan s.n.* (ERE)	LT978528/ LT981270/ LT984420/ LT984453
ALT (1)	H4	Armenia, Lori Province, track from Dashtadem to Tashir, *Tamanyan et al. 07-1189* (ERE)	LT978527/ LT981269/ LT984419/ LT984452
GJP (1)	H4	Georgia, Javakheti, between Aspara and Vladimirovka villages, *Shvanova s.n.* (LE)	LT978526/ LT981268/ LT984418/ LT984451
GBB (1)	H8	Georgia, Borjomi, Bakuriani Botanical Garden, *Merello s.n.* (cult.)	LT978543/ LT981285/ LT984435/ LT984468
LAS (1)	H5	Austria, Niederösterreich, Haltestelle Stillfried, *Barta s.n.* (ERE)	LT978544/ LT981286/ LT984436/ LT984469
Sc1 (1)	H9	United Kingdom, Cambridge University Botanic Garden, *Boltenkov s.n.* (cult.)	LT978558/ LT981300/ LT984450/ LT984482
Sc2 (1)	H5	United Kingdom, Hertfordshire, St. Albans, *Boltenkov s.n.* (cult.)	LT978540/ LT981282/ LT984432/ LT984465
ZOR (1)	H4	Armenia, Zorakert, *Fayvush et al. 09-1696* (ERE)	*LT627901*/ LT628017/ LT628028/ LT628007
Outgroup specimens			
*I.* ser. *Laevigatae*			
*I. ensata*			
ZAR		Russia, Primorsky Krai, Zarubino, *Boltenkov s.n.* (VBGI)	*LT627896/ LT628012/ LT628022/ LT628002*
*I. laevigata*			
ROS		Russia, Primorsky Krai, Roshchino, *Pshennikova s.n.* (cult.)	*LT627897/ LT628013/ LT628024/ LT628003*
*I. pseudacorus*			
VLA		Russia, Vladivostok, *Boltenkov s.n.* (cult.)	*LT627898/ LT628014/ LT628025/ LT628004*
*I.* ser. *Lacteae*			
*I. lactea*			
ZAB		Russia, Zabaykalsky Krai, Kharanor, *Chernova s.n.* (IRK)	*LT627854/*LN871708/ LN871662*/ LN871625*
*I. oxypetala*			
SHI		China, Shaanxi, Suyde, *Kabanov s.n.* (LE)	*LT627844/ LT627950/ LT627975/ LT627911*
*I. tibetica*			
QHU		China, Qinghai, Riyue Xiang, *Long et al. 60* (E)	*LT627892/ LT627943/ LT627997/ LT627932*
*I.* ser. *Ruthenicae*			
*I. uniflora*			
ANIS		Russia, Primorsky Krai, Anisimovka, *Orlovskaya s.n.* (VBGI)	*LT627832*/*LN871684/ LN871640/ LN871604*
ZKY		Russia, Kyrinsky District, *Vologdina s.n.* (cult.)	*LT627902/ LT628018/ LT628029/ LT628008*
*I.* subgen. *Pardanthopsis*			
*I. dichotoma*			
RDA		Russia, Amur Oblast, *Baranova s.n.* (cult.)	LT978555/ LT981297/ LT984447/ LT984483

**Notes.**

N, number of analyzed individuals; H, haplotype; cult., cultivated. * Herbarium codes according to Thiers, 2020. Accession numbers in italics are reported in a previous study (Boltenkov et al., 2018).

DNA extraction, amplification, and direct sequencing of four non-coding cpDNA regions (*trn*S–*trn*G, *trn*L*–trn*F, *rps*4*–trn*S^*GGA*^, and *psb*A–*trn*H) follows Kozyrenko et al. (2004); Kozyrenko, Artyukova & Zhuravlev (2009). The cycle sequencing was accomplished on both strands and fragments were separated using a genetic analyzer ABI 3130 (Applied Biosystems, USA) in the Instrumental Centre of Biotechnology and Gene Engineering (Vladivostok, Russia). Sequences were deposited in the European Nucleotide Archive database; their accession numbers are available in Table 2.

### Data analysis

The sequences of each cpDNA region obtained in this study and retrieved from the complete chloroplast sequence of *I. sanguinea* (KT626943) were aligned manually using the program SeaView v. 4 (*Gouy, Guindon & Gascuel, 2010*) and concatenated for each specimen. We included in the dataset indels and length variation in mononucleotide repeats because repeatability tests allowed us to exclude PCR errors. The haplotypes were identified based on combined DNA sequences using DnaSP v. 5 (*Librado & Rozas, 2009*). This program was also used to calculate the degree of divergence between cpDNA sequences based on nucleotide substitutions. A haplotype network was built using Network v. 4.6 (*Bandelt, Forster & Röhl, 1999*), treating each deletion/insertion, regardless of size as a single mutational event and using the median joining (MJ) algorithm with default settings. To reveal relationships between *I. sanguinea*, *I. sibirica*, and *I*. *typhifolia*, a haplotype network was also built using a dataset including *psb*A–*trn*H and *trn*L–*trn*F sequences obtained in our study and sequences of *I. typhifolia* retrieved from GenBank.

Phylogenetic analyses were performed on two datasets of combined sequences for four cpDNA regions studied (available at https://purl.org/phylo/treebase/phylows/study/TB2:S26635). The first one was composed of sequences from the *I*. subser. *Sibiricae* specimens obtained in the present study, haplotypes of seven taxa of *I*. sect. *Limniris* and *I*. *dichotoma* as outgroup. The second dataset was enlarged by the addition of *psb*A–*trn*H and/or *trn*L–*trn*F sequences for 13 accessions of the *I*. subser. *Sibiricae* species available in GenBank, and for these accessions, lacking portions of sequences (*trn*S–*trn* G and *rps*4–*trn*S regions) were coded as missing. Phylogenetic analyses were performed using Maximum Likelihood (ML) and Maximum Parsimony (MP) methods as implemented in PAUP v. 4.0b10 (*Swofford, 2003*). Bayesian Inference (BI) was conducted using MrBayes v.3.2.6 (*Ronquist & Huelsenbeck, 2003*) on the CIPRES portal (http://www.phylo.org/; *Miller, Pfeiffer & Schwartz, 2010*). For the MP analyses, gaps were coded according to (Simmons & Ochoterena, 2000), as implemented in the program FastGap v. 1.2 (*Borchsenius, 2009*). Optimal trees were found using a heuristic search with 1,000 random addition sequence replicates, starting trees obtained via stepwise addition, tree bisection and reconnection (TBR) branch swapping and the MulTrees option in effect. For ML and BI analyses, GTR + I + G model was selected according to the Akaike information criterion (AIC) using Modeltest v. 3.6 (Posada & Crandall, 1998). ML heuristic searches were done using the resulting model settings, 100 replicates of random sequence addition, TBR branch swapping and MULTrees option on. In BI, using the default prior settings, two parallel MCMC runs were carried out for ten million generations, sampling every 1,000 generations for a total of 10,000 samples. Convergence of the two chains was assessed, and the posterior probabilities (PP) were calculated from the trees sampled during the stationary phase. The robustness of nodes in ML and MP trees was tested using bootstrap with 1,000 replicates (bootstrap percentage, BP).

## Results

### Morphological data

**Figure 3:**
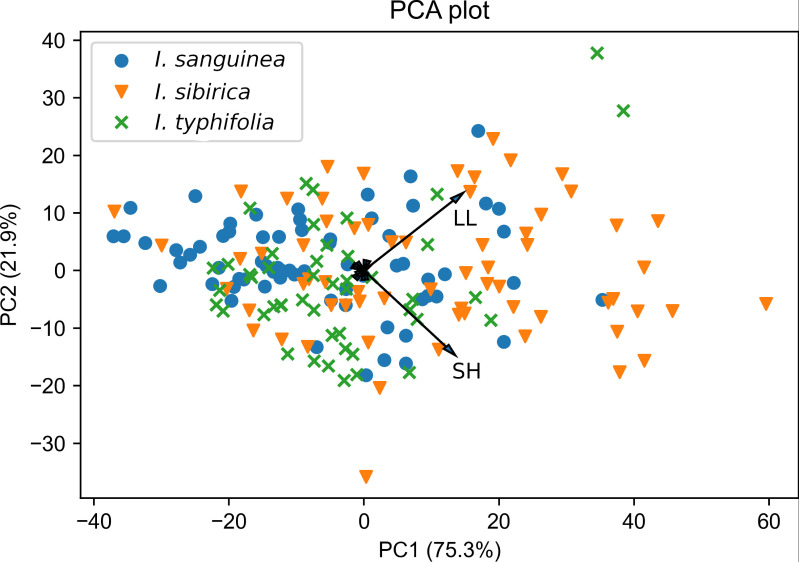
Principal components analysis of the *Iris* subser. *Sibiricae* species based on nine morphological characters. Refer to Table 1 for character abbreviations.

Morphological comparison among the *I.* subser. *Sibiricae* species is provided in Table 3. The results showed overlap of *I. sanguinea*, *I. sibirica*, and *I*. *typhifolia* at the morphological level (Fig. 3, Table 3). The majority of characters were variable in this analysis (see Coefficient of variation in Table S2).

**Table 3:** **Morphological comparison among the*Iris*subser.*Sibiricae*** species.

**Character (code)**		***I. sanguinea***	***I. sibirica***	***I. typhifolia***
Rosette leaf length, cm (LL)		24–77	24–88	28–99
Rosette leaf width, cm (LW)		0.2–0.7(1.1)	0.2–0.8(1.1)	0.2–0.4
Flowering stem height, cm (SH)		23–82	22–99	35–74
Inflorescence structure (IS)		terminal head or occasionally with a lateral head	terminal head or with a lateral head	terminal head or occasionally with a lateral head
Number of flowers (NF)		1–3(4)	1–4(6)	1–3(4)
Number of cauline leaves (NC)		(0)1–2(3)	(0)1–2(3)	1–3
Cauline leaf length, cm (UL)		4–13(25)	3.5–13.5	4–9.5
Bract length, cm (BL)		2–7	2.1–5.5	3–6
Pedicel length ,cm (PL)		0.6–6.5	0.4–6	0.5–6
Flower colour (FC)		blue to violet with purple veins	blue to violet with purple veins	violet with purple veins
Fruit length, cm (FL)		1.7–7.7	1.5–4.2	2.3–5.5
Fruit shape (FS)		oblong-ellipsoidal	oblong-ellipsoidal or ellipsoidal	oblong-ellipsoidal
Seed shape (SS)		semirounded or irregular, flat, thin, slightly glossy, brown	semirounded or irregular, flat, thin, slightly glossy, brown	nearly elliptical, flat, thin, slightly glossy, brown

The result of PCA revealed three characters with high factor loadings (*r* ≥ 0.5) on the first three principal components. These are LL, SH and CL (see abbreviations in Table 1). Together, the first three components accounted for 99.2% of the total variation. The first two components explained 75.3% and 21.9% of the total variation, respectively.

The biplot of PCA for all those species illustrates the overlap between all specimens and significant morphological similarity (Fig. 3). Two characteristics SH and LL displayed the highest correlations with the first and second axis (corresponding values are *r* = 0.73 and *r* = 0.67), and the remaining one (CL) highly influenced the third axis (*r* = 0.93). Results of parametric and non-parametric ANOVA analysis to projected data on three principal components showed that mean (median in case of the non-parametric test) values do not differ significantly among the species. Corresponding statistics and *p*-values are: *p*-value = 0.21 and adjusted *p*-value = 0.63 for traditional ANOVA; *p*-value = 0.03 and adjusted *p*-value = 0.11 for Kruskal–Wallis test. However, being applied to the original plant characters, both parametric and non-parametric ANOVA tests showed significant differences of average values for *I sanquinea*, *I. sibirica*, and *I. typhifolia*. Our results showed that mean (in case of traditional ANOVA) and median (in case of Kruskal–Wallis test) values only for LL and possibly PL do not significantly differ among the considered species (Table S2). Thus, having likely different average values of morphometric characters, caused by environmental conditions and interspecific trait variability, these species can still be considered as indistinguishable in a generalized (PCA) factor space.

### Molecular data

Among the 44 specimens studied, nine haplotypes (H1–H9) were identified based on nucleotide substitutions and indels detected across 3766 aligned positions of four cpDNA regions (Table 2). Four haplotypes (H6–H9) were unique, i.e., found in a single population: H6 in RLV population (Leningrad Oblast, Russia), H7 in population RMS from the Setun River valley (Moscow Oblast, Russia), H8 in population GBB from Georgia, while H9 was found in the plant Sc1 cultivated at the Botanic Garden of Cambridge University, the United Kingdom (UK). Five other haplotypes were detected in more than one accession, often from geographically distant locations in the *I.* subser. *Sibiricae* distribution range. The sequences of cpDNA regions obtained in our study were compared with those from the complete chloroplast sequence of *I. sanguinea* from the Republic of Korea (KT626943). Haplotype H1 found in accessions from two localities in Russia (RP1, RKY) and from three localities in Mongolia (BAD, MDB, and MKB), turned out to be identical with the haplotype of *I. sanguinea* from the Republic of Korea (KT626943). Specimens of populations RP3, RP4, and RP5 from Primorsky Krai, Russia shared haplotype H3, while populations ORL, RP2 (Primorsky Krai) and RCH (Amur Oblast, Russia) shared haplotype H2. Specimens from populations ALS, ALT, and ZOR (Armenia), GJP (Georgia), RKT (Karachay-Cherkess Republic, Russia), RRU (Udmurt Republic, Russia), and RKP (Kurgan Oblast, Russia) shared haplotype H4. Haplotype H5 was found in samples RKU (Kaluga Oblast, Russia), RPS (Pskov Oblast, Russia), and LAS (Austria) as well as in a cultivated plant Sc2 (UK). No specimen from the European part of the distribution range shared haplotypes with plants from the Asian part. The sequence divergence of cpDNA between plants from the European and Asian parts of the distribution range was very low (*K*_S_ = 0.00056).

**Figure 4:**
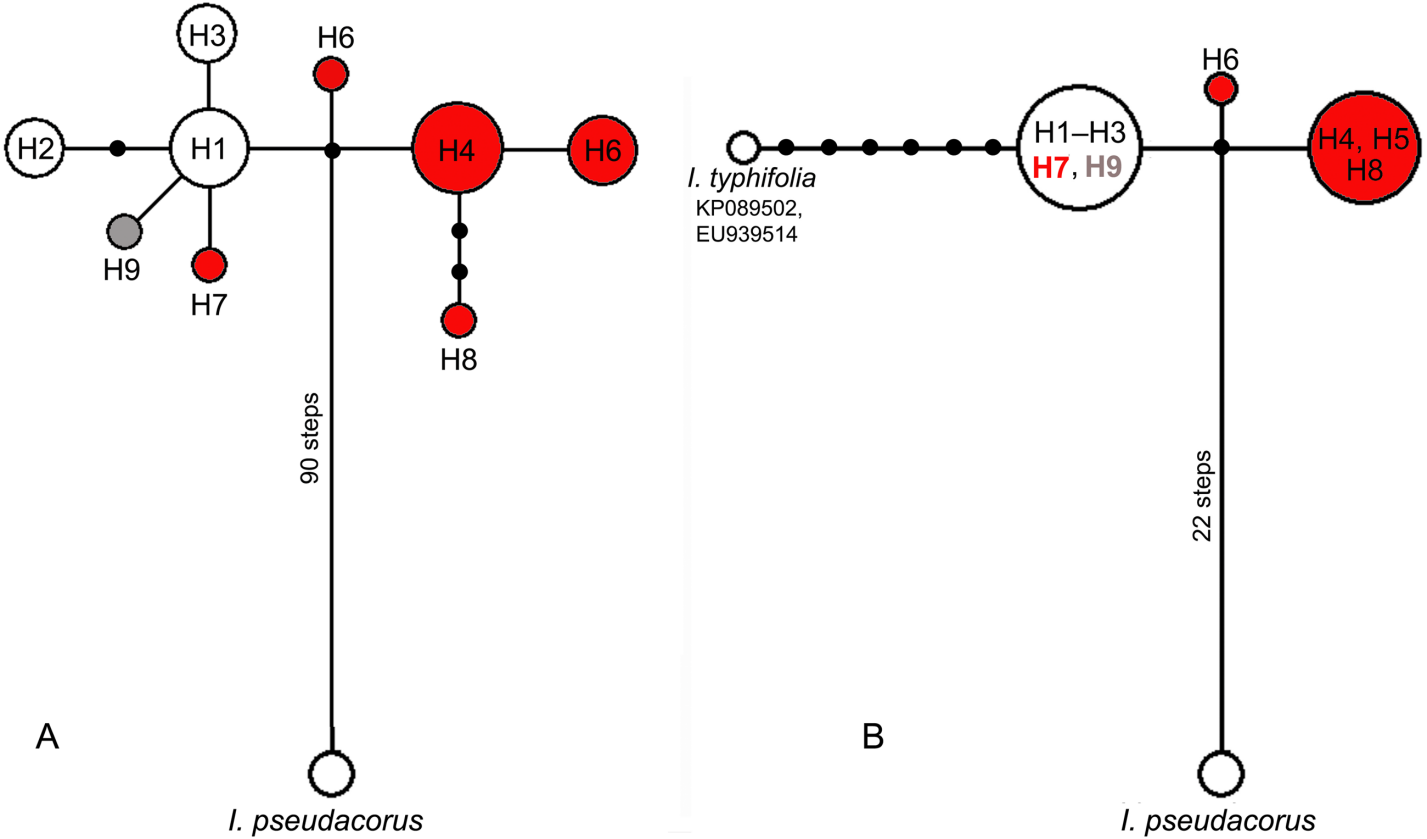
Median-joining networks showing the relationships among cpDNA haplotypes of the *Iris* subser. *Sibiricae* species found in 27 localities across the distribution range including *I. sanguinea* sample from the Republic of Korea (KT626943) and *I. pseudacorus* as outgroup. (A) The data are based on combined sequences of the *trn*S–*trn*G, *trn*L*–trn*F, *rps*4*–trn* S^*GGA*^, and *psb*A–*trn*H regions. (B) The data are based on combined sequences of the *psb*A–*trn*H and *trn*L–*trn*F regions including sequences of *I*. *typhifolia* retrieved from GenBank (KP089502, EU939514). Each circle represents a haplotype and the size of the circle is proportional to the number of population where that haplotype is found. Red circles –haplotypes found in plants from the *I. sibirica* distribution range; white circles –haplotypes found in plants from the *I. sanguinea* distribution range; grey circle –haplotype from cultivated plant S1. Black dots indicate intermediate haplotypes not observed in the sampling. Haplotype codes as in Table 2.

In the median network, all haplotypes formed one group (Fig. 4A) with a minimal divergence between each other (one to three mutational steps). Five haplotypes (H1–H3, H7, and H9) formed a star-like structure with haplotype H1 in the centre. This group composed of all haplotypes (H1–H3) from East Asian plants also included H7 from Eastern Europe and differed only by one substitution in the *psb*A–*trn*H region from all other haplotypes found in plants from the European range, namely haplotypes H4–H6 and H8. All haplotypes found across the *I*. subser. *Sibiricae* distribution range were closely related and derived from the same unsampled or extinct ancestral haplotype connected by many mutation steps with the haplotype of *I*. *pseudacorus* from *I*. ser. *Laevigatae* (Fig. 4A). A similar pattern was obtained in the network based on sequence data from the *psb*A–*trn*H and *trn*L–*trn*F regions, which included sequences of *I. typhifolia* retrieved from GenBank (Fig. 4B). In this network, all specimens from the Asian part of range share the common haplotype connected by six mutational steps with haplotype of *I. typhifolia* and by two steps with two haplotypes found in specimens from the European range.

**Figure 5:**
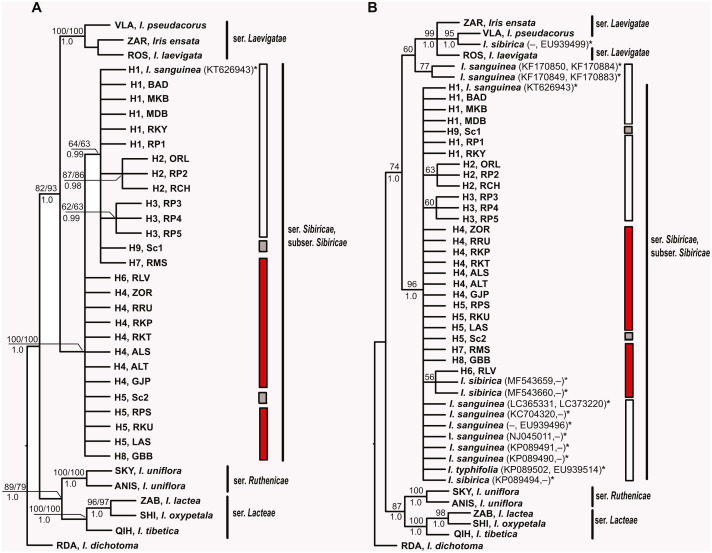
Phylogenetic analysis of *Iris* subser. *Sibiricae*. (A) Strict consensus tree of the six equally most parsimonious trees resulting from MP analysis of combined plastid *trn* S–*trn*G, *trn*L*–trn*F, *rps*4*–trn*S^*GGA*^, and *psb*A–*trn*H sequences from 27 localities across the distribution range of *Iris* subser. *Sibiricae* including *I. sanguinea* sample from the Republic of Korea, KT626943 (Tree length of 429 steps, CI = 0.8228, RI = 0.8905). (B) Strict consensus tree of more than 600,000 equally most parsimonious trees resulting from MP analysis of the enlarged dataset including *psb*A–*trn*H and/or *trn*L–*trn*F sequences for 13 additional accessions of the *I.* subser. *Sibiricae* species retrieved from GenBank (Tree length of 469 steps, CI = 0.7655, RI = 0.8579). The numbers above and below branches indicate bootstrap values (> 50%) for MP/ML analyses and Bayesian posterior probabilities (>0.90) for BI analysis, respectively. Haplotype and locality codes correspond to those in Table 2. The asterisk (*) indicates species names and accession numbers of the sequences retrieved from GenBank. Bars indicate the geographical origin of the examined populations: white –East Asia; red –Europe and Western Siberia; grey –cultivated plants.

MP, ML and BI analyses based on sequences of *I*. subser. *Sibiricae* obtained in the present study yielded similar topologies with few differences in node statistical supports (Fig. 5A). All *Iris* specimens clustered into highly supported (BP 100, 100%, PP 1.0) clades according to their affiliation to corresponding series of *I*. sect. *Limniris*. Haplotypes of all plants belonging to *I.* subser. *Sibiricae* formed a monophyletic highly supported clade (BP 100, 100%, PP 1.0) sister to the clade including species of *I.* ser. *Laevigatae* (BP 82, 93%, PP 1.0). Within the *I.* subser. *Sibiricae* clade, it was possible to distinguish a group including haplotypes H1–H3 from the Asian part of range, haplotype H7 from the Moscow Oblast (Russia), as well as haplotype H9 of the cultivated plant (Sc1), though this group received poor support in the MP and ML analyses (BP 63, 64%) and strong support only in BI analysis (PP 0.99). The overall topology of MP and BI trees (Fig. 5B) constructed with dataset including thirteen accessions of the *I*. subser. *Sibiricae* species retrieved from GenBank was largely similar to those of the trees described above (Fig. 5A). Ten of the thirteen additional accessions of *I. sanguinea*, *I. sibirica*, and *I. typhifolia* were placed together with all specimens of *I*. subser. *Sibiricae* in a monophyletic group (BP 100%, PP 1.0). However, the phylogenetic relationships within this clade were unresolved. Only one of three *I. sibirica* accessions (voucher *Mosulishvili G99-12*, RSA; see *Wilson, 2009)* and two (isolates ISD1 and ISD2, *Lee & Park, 2013)* of six accessions of *I. sanguinea* from the Republic of Korea were placed outside of the *I*. subser. *Sibiricae* clade but clustered with the *I.* ser. *Laevigatae* species (Fig. 5B). The sequence divergence (*K*_S_) calculated for two cpDNA regions between Korean accessions of *I. sanguinea* placed in the *I*. ser *Laevigatae* clade and *I. sanguinea* accessions placed in the *I*. subser *Sibiricae* clade was 0.009510 that was comparable with divergence between species in other series of *I.* sect *Limniris* (0.00451–0.01223; *Boltenkov et al., 2018)*.

## Discussion

The overlapping of some previously considered diagnostic characters of *I. sanguinea*, *I. sibirica*, and *I*. *typhifolia* (see Fig. 3, Table 3) indicates that they constitute a group of morphologically very similar taxa, difficult to tell apart. We came to the conclusion that the key characters reported to distinguish *I. typhifolia* from *I. sibirica* are not stable and overlap among specimens attributed to either name.

Our examination of herbarium specimens and the analysis of the relevant literature revealed a wide range of variation in *I. sanguinea* and *I. sibirica* morphological characters. Key morphological characters discriminating *I. sanguinea* and *I. sibirica* are considered the features of the flowering stem structure. However, our data show that the flowering stems can be longer or shorter than the basal leaves, depending on the phenological phase, as well as simple or branched (Table 3). *Skrypec & Odintsova (2017)* also reported a high variability of the *I. sibirica* inflorescences structure. In our survey of herbarium specimens from the *I.* subser. *Sibiricae* distribution range, most plants had a flowering stem with terminal head of two flowers. In some parts of the *I.* subser. *Sibiricae* distribution range, plants with terminal and one lateral head are rarer (i.e., Omsk Oblast, Novosibirsk Oblast, and Buryatia Republic) or are the only ones (northern Kazakhstan, north of the European part of the Russia, Irkutsk Oblast, Zabaykalsky Krai, Sakha Republic, and Russian Far East). Previously, Poljakov (1958) indicated that the plants with terminal head is the typical of *I. sibirica* in northern Kazakhstan. Therefore, contrary to the general assumption of many botanists, inflorescence structure could not be a diagnostic key to distinguish species in *I.* subser. *Sibiricae*. In addition, our data showed that leaf width is variable in both *I. sanguinea* and *I. sibirica*, so it could not be used as a diagnostic character either. Differences of these characters observed may be the result of environmental conditions and the variability of characters within the species.

In the present study, we also failed to genetically distinguish between specimens collected in different localities of the *I.* subser. *Sibiricae* distribution range where *I. sanguinea* or *I. sibirica* are considered to occur (Figs. 4 and 5). Our analyses based on sequence variability in four non-coding regions of cpDNA showed an absence of clear differentiation between plants of *I. sanguinea* growing eastward Lake Baikal and *I. sibirica* distributed in Europe and Western Siberia*.* All specimens studied were closely related to each other and are clearly separated from other species in *I*. sect. *Limniris*. However the samples from the *I. sanguinea* distribution range together with a specimen RMS from European part of the range formed a distinct clade supported only in BI analysis (Fig. 5). Only one single point mutation in *psb* A–*trn* H distinguished these groups indicating their minimally differentiation. Nucleotide divergence of cpDNA between these groups (*K*_S_ = 0.00056) is lower than between species in other series of *I.* sect. *Limniris* (0.00451–0.01223; *Boltenkov et al., 2018*) and comparable with divergence between populations of some *Iris* species, e.g., *I. lactea* (0.00037–0.00112; *Boltenkov, Artyukova & Kozyrenko, 2016*). The star-like structure of haplotype diversity also indicates an absence of deep phylogenetic split between plants from European and Asian parts of the *I*. subser. *Sibiricae* distribution range and is consistent with a rapid range expansion (*Ferreri, Qu & Han, 2011*).

In phylogenetic trees (Figs. 5A, 5B), all 44 specimens of Siberian irises studied as well as most accessions of *I.* subser. *Sibiricae* available in GenBank (including *I. typhifolia*) form a single monophyletic clade sister to the clade including species of *I.* ser. *Laevigatae*. Previously, the monophyly of the *I.* subser. *Sibiricae* species was also shown in phylogenetic study of Tillie, Chase & Hall (2000). In other studies, where the same one specimen (voucher *Mosulishvili G99-12*, RSA) was used as sole representative of *I. sibirica*, this specimen was embedded within the clade comprising species from *I.* ser. *Laevigatae* (*Wilson, 2009; Mavrodiev et al., 2014*) or *I*. ser. *Lacteae* (*Jiang et al., 2018*), thus making *I.* subser. *Sibiricae* polyphyletic. Crespo, Martínez-Azorín & Mavrodiev (2015) have pointed out that additional samples of *I. sibirica* should be sequenced to determine the phylogenetic position of this species at the infrageneric level. The specimen *Mosulishvili G99-12* was confirmed as a misidentification (Carol Wilson & Marine Mosulishvili, 2020, pers. comm.). Only DNA material, but no herbarium voucher was collected by Mosulishvili from Kazbegi, north-eastern Georgia, in 1999. Moreover, it was noted (Mosulishvili, 2020, pers. comm.), that *I. sibirica* was never found near Kazbegi, while *I. pseudacorus* is common in this area. Other two samples of *I.* subser *Sibiricae* (isolates ISD1 and ISD2, *Lee & Park, 2013)* that had fallen into the clade of the *I*. ser *Laevigatae* species were of *I*. *sanguinea* from the Republic of Korea. Large divergence of these samples from all other samples of *I*. *sanguinea* from the Republic of Korea and other parts of the distribution range is comparable with divergence between different species of *I*. sect *Limniris* and the further studies are required to establish the species affiliation of these Korean samples. In this work, none of the studied specimens belonging to *I.* subser. *Sibiricae* fell within the *I.* ser. *Laevigatae* clade. Thus, our results clearly show that *I*. subser. *Sibiricae* is a monophyletic taxon that is strongly supported as sister to the *I.* ser. *Laevigatae* species.

The broad morphological variation, including inflorescence structure, observed in the group surveyed, together with the molecular results, point out to the difficulty in separating *I. sanguinea* at specific rank. Evidently, *I. sibirica* includes a set of morphotypes, but it remains homogeneous taxonomically, without possible recognition of infraspecific taxa or separate species, as evidenced by the molecular data obtained in this study. Therefore, we regard *I. sanguinea*, *I. sibirica*, and *I*. *typhifolia* as synonymous and formally propose a reduction of *I. sanguinea* and *I*. *typhifolia* to *I. sibirica*, which is the earliest legitimate name and has priority (Art. 11.3, Turland et al., 2018).

### Taxonomic treatment

In the present study we confirm that *I.* subser. *Sibiricae* includes only a single variable species, *I. sibirica*. It is the most widespread *Iris* species, occurring from Central and Eastern Europe, including northeast Turkey, northern Kazakhstan, and Caucasus, to Siberia, East Asia (northern Mongolia, northern and eastern China, Korean Peninsula, and Japan), and the southern Russian Far East. It is found growing wild in moist meadows along river valleys. It is cultivated worldwide and sometimes naturalized. Morphologically, *I. sibirica* is distinct from *I*. subser. *Chrysographes* species by having shorter bracts (2–6 cm long), a much shorter perianth tube (no more than 0.5 cm long), and green basal leaves. The synonymic list of taxa specified in the present work, including types, is provided below.

***Iris sibirica*** L., Sp. Pl. 1: 39. 1753. ≡ *Iris pratensis* Lam., Fl. Franç. 3: 498. 1779, *nom. illeg.* (Art. 52.1, Turland et al., 2018). ≡*Biris sibirica* (L.) Medik., Staatswirthschaftl. Vorles. Churpfälz. Phys.-Ökon. Ges. Heidelberg, 1: 257. 1791. ≡ *Iris stricta* Moench, Methodus, 2: 528. 1794, *nom. illeg.* (Art. 52.1). ≡ *Iris angustifolia* Salisb., Prodr. Stirp. Chap. Allerton: 44. 1796, *nom. illeg.* (Art. 52.1). ≡ *Xiphion sibiricum* (L.) Schrank, Flora 7(2, Beil.): 19. 1824. ≡ *Xiphion pratense* Parl., Nuov. Gen. Sp. Monocot.: 45. 1854. ≡ *Limniris sibirica* (L.) Fuss, Fl. Transsilv.: 637. 1866. ≡ *Xyridion sibiricum* (L.) Klatt, *Bot. Zeitung (Berlin), 30: 500.* 1872. –* Limnirion sibiricum* (L.) Opiz, Seznam: 5. 1852, *nom. inval.* (Art. 38.1). –* Iris sibirica* var. *typica* Maxim., Bull. Acad. Imp. Sci. Saint-Pétersbourg, 26: 519. 1880, *nom. inval .* (Art. 24.3). Type: [Specimen from a cultivated plant]. *sibirica* 9, HU [Horto Upsaliensis], Herb. Linnaeus (lectotype: designated by *Altinordu & Crespo, 2016*: 297, LINN! [LINN No. 61.20]).

*= Iris orientalis* Thunb., Trans. Linn. Soc. London, 2: 328. 1794, *nom. illeg.* (non Mill., Gard. Dict., ed. 8: *Iris* No. 9. 1768; Art. 53.1), ***syn. nov.*** ≡ *Xiphion orientale* Schrank, Flora 7(2, Beil.): 19. 1824. ≡ *Iris sibirica* var. *orientalis* (Schrank) Baker, J. Linn. Soc., Bot. 16: 139. 1877. ≡ *I. extremorientalis* Koidz., Bot. Mag. (Tokyo), 40: 330. 1926, *nom. nov.* (Art. 6.11). Type: Japan. [Note on the upper side]: *Iris sibirica*, Fl. jap. p. 33, Barin; [Note on the reverse side]: e Japonia, *Thunberg s.n.*, Herb. Thunberg (lectotype: UPS [UPS-THUNB 1144, image!], designated here by E.V. Boltenkov).

= *Iris sanguinea* Hornem., Hort. Bot. Hafn. 1: 58. 1813, ***syn. nov.*** ≡ *I. sibirica* var. *sanguinea* (Hornem.) Ker Gawl., Bot. Mag. 39: t. 1604. 1814. ≡ *Limniris sanguinea* (Hornem.) Rodion., Bot. Zhurn. (Moscow & Leningrad), 92: 551. 2007. –* Iris sanguinea* Donn, Hort. Cantabrig., ed. 6: 17. 1811, *nom. inval.* (Art. 38.1) –* I. sanguinea* var. *typica* Makino, J. Jap. Bot. 6: 32. 1930, *nom. inval.* (Art. 24.3). Type: [Specimen from a cultivated plant]. [Handwriting 1]: *Iris sanguinea*, ex hort. bot. Hafn.; [Handwriting 2]: [*Iris sanguinea* ] Don., ad *I. sibir* [*ica* ]. L. ref. spr., Herb. Hornemann (lectotype: designated by *Boltenkov, 2018*: 178, C [C10022296, image!]).

= *Iris sibirica* var. *haematophylla* Besser, Flora, 17(1, Beibl.): 25. 1834, ***syn. nov.*** Type: [Specimen from a cultivated plant]. *Iris* (*sibirica*) *haematophylla*, Dahuria, *Fischer s.n.*, Herb. Lindley (neotype: CGE! [CGE14724 ], designated here by E.V. Boltenkov).

= *Iris typhifolia* Kitag., Bot. Mag. (Tokyo), 48: 94. 1934, *syn. nov.* ≡ *Limniris typhifolia* (Kitag.) Rodion., Bot. Zhurn. (Moscow & Leningrad), 92: 551. 2007. Type: China. [Liaoning Province], *Iris sibirica*? …14 Aug. 3 [1928], *K. Yamatsuta 60* (holotype: TI [image!]).

## Conclusions

In *Iris* subser. *Sibiricae*, both morphological and geographical aspects are important to delimitate species. In this group, *I. sanguinea*, *I. sibirica*, and *I. typhifolia* have been recognized. However, analyses of morphological and molecular phylogenetic data may allow positioning the species among its relatives more exactly. In the case presented here, we reconstructed the phylogeny based on four non-coding regions of plastid DNA (*trn*S–*trn*G, *trn*L*–trn*F, *rps*4*–trn*S^*GGA*^, and *psb*A–*trn*H), and explored morphological characters to determine the relationship between species. At the same time, we once again showed that these regions are very informative for the taxonomy of irises, as they allow identifying species. Our results show that the morphological characters of *I. sanguinea*, *I. sibirica*, and *I. typhifolia* are overlaping. Phylogeny studies show that in accordance with the current circumscription, *Iris* subser *Sibiricae* is not polyphyletic. All the three species are nested together forming a well-supported monophyletic group (BP 100%, PP 1.0). It is thus concluded that *I. sanguinea* and *I*. *typhifolia* are conspecific with *I. sibirica*, a previously described species.

##  Supplemental Information

10.7717/peerj.10088/supp-1 Supplemental Information 1 Sample information of the *Iris* subser. *Sibiricae* accessions from GenBank A dash (–) indicates not available.

10.7717/peerj.10088/supp-2 Supplemental Information 2 The results of the variance analysis of the *Iris* subser. *Sibiricae* species M –mean (cm), S –standard deviation, C –coefficient of variation (%). The values in parentheses are adjusted *p*-values. Refer to Table 1 for character abbreviations.

10.7717/peerj.10088/supp-3 Supplemental Information 3 Complete list of specimens examined in the morphological study

10.7717/peerj.10088/supp-4 Supplemental Information 4 Raw data of the morphological analysis

## Additional Information and Declarations

### DNA Deposition

The following information was supplied regarding the deposition of DNA sequences: All sequences described here are available at GenBank: LT978526 to LT978545 and LT978555 to LT978558 for *trn*H–*psb*A, LT981268 to LT981287 and LT981297 to LT981300 for *rps*4–*trn*S, LT984418 to LT984437 and LT984447 to LT984450 for *trn*S–*trn*G, LT984451 to LT984470 and LT984480 to LT984483 for *trn*L–*trn*F (see Table 2). To reveal relationships between *I. typhifolia*, *I. sibirica* and *I. sanguinea*, a haplotype network was also built using a dataset including *psb*A–*trnH* and *trn*L–*trn*F sequences obtained in our study and sequences of *I. typhifolia* from GenBank.

### Data Availability

The following information was supplied regarding data availability: The raw measurements are available in the Supplementary Files.

## References

[ref-1] Altinordu F, Crespo MB (2016). Nomenclatural type designation of four Linnaean names in *Iris sensu lato* (Iridaceae). Phytotaxa.

[ref-2] Baker JG (1877). Systema Iridacearum. The Journal of the Linnean Society, Botany.

[ref-3] Bandelt H-J, Forster P, Röhl A (1999). Median-joining networks for inferring intraspecific phylogenies. Molecular Biology and Evolution.

[ref-4] Benjamini Y, Hochberg Y (1995). Controlling the false discovery rate: a practical and powerful approach to multiple testing. Journal of the Royal Statistical Society, Series B.

[ref-5] Boltenkov EV, Artyukova EV, Kozyrenko MM (2016). Species divergence in *Iris* series *Lacteae* (Iridaceae) in Russia and adjacent countries based on chloroplast DNA sequence data. Russian Journal of Genetics.

[ref-6] Boltenkov EV (2018). Typification of the name *Iris sanguinea* (Iridaceae). Phytotaxa.

[ref-7] Boltenkov EV, Artyukova EV, Kozyrenko MM, Trias-Blasi A (2018). *Iris tibetica*, a new combination in *I*. ser. *Lacteae* (Iridaceae) from China: evidence from morphological and chloroplast DNA analyses. Phytotaxa.

[ref-8] Borchsenius F (2009). FastGap 1.2.

[ref-9] Crespo MB, Martínez-Azorín M, Mavrodiev EV (2015). Can a rainbow consist of a single colour? A new comprehensive generic arrangement of the ‘*Iris sensu latissimo*’ clade (Iridaceae), congruent with morphology and molecular data. Phytotaxa.

[ref-10] Dénes A, Juhász M, Salamon-Albert É (2008). A szibériai nöszirom (*Iris sibirica* L.) egy Dráva menti állományának változásai 2000–2007 között. Somogyi Múzeumok Közleményei.

[ref-11] Diels L, Engler A, Prantl K (1930). Iridaceae. Die natürlichen Pflanzenfamilien, 2nd edition, 15a.

[ref-12] Dodge Y (2008). The concise encyclopedia of statistics.

[ref-13] Doronkin VM, Malyshev LI, Peschkova GA (1987). Iridaceae. Flora of Siberia, vol. 4.

[ref-14] Doronkin VM, Baikov KS (2012). Iridaceae Juss. Synopsis of flora of Asian Russia: vascular plants.

[ref-15] Dykes WR (1910). Three new Chinese irises. The Gardeners’ Chronicle, series 3.

[ref-16] Dykes WR (1912). The genus *Iris*.

[ref-17] Fedtschenko BA, Komarov VL (1935). Iris. Flora of the USSR, vol. 4.

[ref-18] Fedtschenko BA, Shishkin BK, Dorozhkin NA (1949). Iridaceae. Flora of the Byelorussian S.S.R., vol. 1.

[ref-19] Ferreri M, Qu W, Han B (2011). Phylogenetic networks: a tool to display character conflict and demographic history. African Journal of Biotechnology.

[ref-20] Galanin AV (2009). Flora of Dahuria, vol. 2.

[ref-21] Global Biodiversity Information Facility (2020). GBIF occurrence download. https://doi.org/10.15468/dl.yc3yg8.

[ref-22] Gouy M, Guindon S, Gascuel O (2010). SeaView version 4: a multiplatform graphical user interface for sequence alignment and phylogenetic tree building. Molecular Biology and Evolution.

[ref-23] Grey-Wilson C (1971). The genus Iris, subsection Sibiricae.

[ref-24] Grey-Wilson C, The Species Group of the British Iris Society (2012). Series *Sibiricae* (Diels) Lawrence. A guide to species irises: their identification and cultivation.

[ref-25] Grubov VI, Grubov VI (1977). Iridaceae. Plants of Central Asia, vol. 7.

[ref-26] Guo J, Wilson CA (2013). Molecular phylogeny of crested *Iris* based on five plastid markers (Iridaceae). Systematic Botany.

[ref-27] Hooker JD (1899). Iris delavayi. Curtis’s Botanical Magazine.

[ref-28] Hornemann JW (1813). Hortus regius botanicus Hafniensis, in usum tyronum et botanophilorum, vol. 1.

[ref-29] Hu H, Al-Shehbaz IA, Sun Y, Hao G, Wang Q, Liu J (2015). Species delimitation in *Orychophragmus* (Brassicaceae) based on chloroplast and nuclear DNA barcodes. Taxon.

[ref-30] Jiang Y-L, Huang Z, Liao J-Q, Song H-X, Luo X-M, Gao S-P, Lei T, Jiang M-Y, Jia Y, Chen Q-B, Yu X-F, Zhou Y-H (2018). Phylogenetic analysis of *Iris* L. from China on chloroplast *TRN*L-F sequences. Biologia.

[ref-31] Kitagawa M (1934). Contributio ad cognitionem florae Manshuricae III. Botanical Magazine.

[ref-32] Koidzumi G (1926). Contributiones ad cognitionem florae Asiae Orientalis. Botanical Magazine.

[ref-33] Komarov VL (1901). Flora Manshuriae. Trudy Imperatorskogo S.-Peterburgskogo Botanicheskogo Sada.

[ref-34] Kozyrenko MM, Artyukova EV, Boltenkov EV, Lauve LS (2004). Somaclonal variability of *Iris pseudacorus* L. according to RAPD and cytogenetic analyses. Biotechnology in Russia.

[ref-35] Kozyrenko MM, Artyukova EV, Zhuravlev YUN (2009). Independent species status of *Iris vorobievii* N.S.Pavlova, *Iris mandshurica* Maxim. and *Iris humilis* Georgi (Iridaceae): evidence from the nuclear and chloroplast genomes. Russian Journal of Genetics.

[ref-36] Krylov PN, Krylov PN (1929). Iridaceae. Flora of Western Siberia, vol. 3.

[ref-37] Kuhn M, Johnson K (2018). Applied predictive modeling.

[ref-38] Lawrence GHM (1953). A reclassification of the genus *Iris*. Gentes Herbarum.

[ref-39] Ledebour CF (1852). Flora Rossica sive enumeration plantarum in totius imperii rossici provinciis europaeis, asiaticis et americanis hucusque observatarum, vol. 4.

[ref-40] Lee H-J, Park SJ (2013). A phylogenetic study of Korean *Iris*L. based on plastid DNA (*psb*A–*trn*H, *trn*L–F) sequences. Korean Journal of Plant Taxonomy.

[ref-41] Lee H-J, Nam G-H, Kim K, Lim CE, Yeo J-H, Kim S (2017). The complete chloroplast genome sequences of *Iris sanguinea* Donn ex Hornem. Mitochondrial DNA.

[ref-42] Lenz LW (1976). A reclassification of the Siberian irises. Aliso.

[ref-43] Librado P, Rozas J (2009). DnaSP v5: a software for comprehensive analysis of DNA polymorphism data. Bioinformatics.

[ref-44] Linnaeus C (1753). Species plantarum.

[ref-45] Liu J, Provan J, Gao L-M, Li D-Z (2012). Sampling strategy and potential utility of indels for DNA barcoding of closely related plant species: a case study in *Taxus*. International Journal of Molecular Sciences.

[ref-46] Löve A (1975). IOPB chromosome number reports XLVII. Taxon.

[ref-47] Mathew B (1989). The Iris.

[ref-48] Mavrodiev EV, Martínez-Azorín M, Dranishnikov P, Crespo MB (2014). At least 23 genera instead of one: The case of *Iris* L. s.l. (Iridaceae). PLOS ONE.

[ref-49] Maximowicz CJ (1880). Diagnoses plantarum novarum asiaticarum. III. Bulletin de l’Académie Impériale des Sciences de Saint-Pétersbourg.

[ref-50] McEwen C, McGarvey W, Warburton B, Hamblen M (1978). Siberian irises. The world of irises.

[ref-51] Miller MA, Pfeiffer W, Schwartz T (2010). Creating the CIPRES Science Gateway for inference of large phylogenetic trees. Proceedings of the Gateway Computing Environments Workshop (GCE).

[ref-52] Pavlova NS, Kharkevich SS (1987). Iridaceae Juss. Vascular plants of the Soviet Far East, vol. 2.

[ref-53] Pedregosa F, Varoquaux G, Gramfort A, Michel V, Thirion B, Grisel O, Blondel M, Prettenhofer P, Weiss R, Dubourg V, Vanderplas J, Passos A, Cournapeau D, Brucher M, Perrot M, Duchesnay É (2011). Scikit-learn: machine learning in Python. Journal of Machine Learning Research.

[ref-54] Poczai P, Hyvönen J (2010). Nuclear ribosomal spacer regions in plant phylogenetics: problems and prospects. Molecular Biology Reports.

[ref-55] Poljakov PP, Pavlov NV (1958). Iris. Flora of Kazakhstan, vol. 2.

[ref-56] Posada D, Crandall KA (1998). Modeltest: testing the model of DNA substitution. Bioinformatics.

[ref-57] Probatova NS (2006). Chromosome numbers of plants of the Primorsky Territory, the Amur River basin and Magadan region. Botanicheskii Zhurnal.

[ref-58] Regel E (1867). Index seminum, quae hortus botanicus imper. Petropolitanus pro.

[ref-59] Rodionenko GI (2007). On the independence of the genus *Limniris* (Iridaceae). Botanicheskii Zhurnal.

[ref-60] Ronquist F, Huelsenbeck JP (2003). MrBAYES3: Bayesian phylogenetic inference under mixed models. Bioinformatics.

[ref-61] Sergievskaya LP (1972). Flora of Transbaikal, vol. 4.

[ref-62] Simmons MP, Ochoterena H (2000). Gaps as characters in sequence-based phylogenetic analyses. Systematic Biology.

[ref-63] Simonet M (1934). Nouvelles recherché cytologiques et génétiques chez les *Iris*. Annales des Sciences Naturelles; Botanique, series 10.

[ref-64] Skrypec CH, Odintsova A (2017). Morphological structure of the inflorescence in *Gladiolus inbricatus* L. and *Iris sibirica* L. (Iridaceae). Studia Biologica.

[ref-65] Spach E (1846). Histoire Naturelle des Végétaux. Phanerogames, vol. 13.

[ref-66] Swofford DL (2003).

[ref-67] Szöllösi R, Medvegy A, Benyes E, Németh A, Mihalik E (2011). Flowering phenology, floral display and reproductive success of *Iris sibirica*. Acta Botanica Hungarica.

[ref-68] Thiers B (2020). Index Herbariorum: a global directory of public herbaria and associated staff. New York Botanical Garden’s Virtual Herbarium. https://sweetgum.nybg.org/ih/.

[ref-69] Tillie N, Chase MW, Hall T (2000). Molecular studies in the genus *Iris* L.: a preliminary study. Annali di Botanica, new series.

[ref-70] Turland NJ, Wiersema JH, Barrie FR, Greuter W, Hawksworth DL, Herendeen PS, Knapp S, Kusber W-H, Li D-Z, Marhold K, May TW, McNeill J, Monro AM, Prado J, Price MJ, Smith GF (2018). International code of nomenclature for algae, fungi, and plants (Shenzhen Code) adopted by the Nineteenth International Botanical Congress Shenzhen, China, 2017. [Regnum Vegetabile 159].

[ref-71] Vicente A, Alonso MA, Crespo MB (2019). *Biscutella pseudolyrata* (Brassicaceae, Biscutelleae), a new species endemic to NW Morocco based on morphological and molecular evidence. Willdenowia.

[ref-72] Virtanen P, Gommers R, Oliphant TE, Haberland M, Reddy T, Cournapeau D, Burovski E, Peterson P, Weckesser W, Bright J, Walt SJ, Brett M, Wilson J, Millman KJ, Mayorov N, Nelson ARJ, Jones E, Kern R, Larson E, Carey CJ, Polat İ, Feng Y, Moore EW, VanderPlas J, Laxalde D, Perktold J, Cimrman R, Henriksen I, Quintero EA, Harris CR, Archibald AM, Ribeiro AH, Pedregosa F, Mulbregt P (2020). SciPy 1.0: fundamental algorithms for scientific computing in Python. Nature Methods.

[ref-73] Waddick JW, Zhao Y-T (1992). Iris of China.

[ref-74] Wheeler AS, Wilson CA (2014). Exploring phylogenetic relationships within a broadly distributed Northern Hemisphere group of semi-aquatic *Iris* species (Iridaceae). Systematic Botany.

[ref-75] Wilson CA (2004). Phylogeny of *Iris* based on chloroplast *matK* gene and *trnK* intron sequence data. Molecular Phylogenetics and Evolution.

[ref-76] Wilson CA (2009). Phylogenetic relationships among the recognized series in *Iris* section *Limniris*. Systematic Botany.

[ref-77] Wilson CA (2011). Subgeneric classification in *Iris* re-examined using chloroplast sequence data. Taxon.

[ref-78] Wilson CA, Padiernos J, Sapir Y (2016). The royal irises (*Iris* subg. *Iris* sect. *Oncocyclus*): plastid and low-copy nuclear data contribute to an understanding of their phylogenetic relationships. Taxon.

[ref-79] Wilson CA (2017). Sectional relationships in the Eurasian bearded *Iris* (subgen. *Iris*) based on phylogenetic analyses of sequence data. Systematic Botany.

[ref-80] Zhao Y-T, Noltie HJ, Mathew B, Wu Z-Y, Raven PH (2000). Iridaceae. Flora of China, vol. 24.

[ref-81] Zheng Y, Meng T, Bi X, Lei J (2017). Investigation and evaluation of wild *Iris* resources in Liaoning Province, China. Genetic Resources and Crop Evolution.

