# Supplementary material for: Iris sanguinea is conspecific with I. sibirica (Iridaceae) according to morphology and plastid DNA sequence data
Source: PeerJ. 2020 Oct 1;8:e10088. doi: 10.7717/peerj.10088 (PMC7533061; doi:10.7717/peerj.10088)
Supplement: Supplemental Information 1 — A dash (–) indicates not available. [file peerj-08-10088-s001.rtf]

Table S1 Sample information of the Iris subser. Sibiricae accessions retrieved from GenBank.
Species	Locality (voucher/isolate)	GenBank accession numbers	Reference	
		trnH–psbA	trnL–trnF		
I. sanguinea	Republic of Korea, Jongja, Pocheon, Gyeonggi-do (NIBRVP0000138241)	KT626943	KT626943	Lee et al., 2017	
I. sanguinea	Japan, Nagano (isolate Isan)	LC365331	–	Yamamoto & Uchida, 2018	
	Japan, Nagano (isolate TBG 147645)	–	LC373220	Mizuno et al., 2018	
I. sanguinea	Republic of Korea, Chuncheon-si (isolate ISD1, KWNU 063104)	KF170849	KF170883	Lee & Park, 2013	
I. sanguinea	Republic of Korea, Busan-si (isolate ISD2, YNUH: LJH03)	KF170850	KF170884	Lee & Park, 2013	
I. sanguinea	Republic of Korea, Janggunbong, Yongmoonsan, Yongmoon-myeon, Yangpyeong-gun, Gyeonggi-do (isolate PDBK2012-0442)	KC704320	–	Hong et al., 
unpublished	
I. sanguinea	Republic of Korea, Yongmoonsan, Saneum-ri, Danwol-myeon, Yangpyeong-gun, Gyeonggi-do (isolate PDBK2012-0413)	KC704319	–	Hong et al., 
unpublished	
I. sanguinea	Republic of Korea, Baegam-san, Waya-ri, Naechon-myeon, Hongcheon-gun, Gangwon-do (isolate PDBK2007-0227)	KC704318	–	Hong et al., 
unpublished	
I. sanguinea	Republic of Korea, Banseon~Dalgung, Sannae-myeon, Namwon-si, Jeollabuk-do (isolate PDBK2006-0704)	KC704317	–	Hong et al., 
unpublished	
I. sanguinea	China (KUN 90487)	JN045011	–	Li et al., 2011	
I. sanguinea	China, Liaoning, Benxi, z109	KP089491	–	Dong et al., 2015	
I. sanguinea	China, Heilongjiang, Yichun, z002 	KP089490	–	Dong et al., 2015	
I. sanguinea	USA, UC (UCBG 93.0838)	–	EU939496	Wilson, 2009	
I. sibirica	USA (RSA)	–	EU939499	Wilson, 2009	
I. sibirica	China, Beijing, Beijing Botanical Garden (CAS)	KP089494	–	Dong et al., 2015	
I. sibirica	Italy, Aosta Valley, Brissogne, Petit-Banc (IRSIBR07-200590)	MF543660	–	Guglielmo et al., unpublished	
I. sibirica	Italy, Aosta Valley, Quart, Olleyes (IRSIQU01-130516)	MF543659	–	Guglielmo et al., unpublished	
I. typhifolia	China, Heilongjiang, Anda, z013
USA (Denver Botanic Garden 022383)	KP089502
–	–
EU939514	Dong et al., 2015
Wilson, 2009	
Note:
A dash (–) indicates not available.

REFERENCES
Dong W, Xu C, Li C, Sun J, Zuo Y, Shi S, Cheng T, Guo J, Zhou S. 2015. ycf1, the most promising plastid DNA barcode of land plants. Scientific Reports 5: 8348 DOI 10.1038/srep08348.
Guglielmo F, Poggio L, Tutino S. Unpublished. DNA barcoding of land plant species in Aosta Valley (Northwest Italy).
Hong J-R, Kim H-W, Kumar K, Kim K-J. Unpublished. DNA sequence based species identification of Asparagales for the Korean flora (psbA-trnH).
Lee H-J, Nam G-H, Kim K, Lim CE, Yeo J-H, Kim S. 2017. The complete chloroplast genome sequences of Iris sanguinea Donn ex Hornem. Mitochondrial DNA 28:15–16 DOI 10.3109/19401736.2015.1106521.
Lee H-J, Park SJ. 2013. A phylogenetic study of Korean Iris L. based on plastid DNA (psbA– trnH, trnL–F) sequences. Korean Journal of Plant Taxonomy 43:227–235. DOI 10.11110/kjpt.2013.43.3.227.
Li DZ, Gao LM, Li HT, Wang H, Ge XJ, Liu JQ, Chen ZD, Zhou SL, Chen SL, Yang JB. 2011. Comparative analysis of a large dataset indicates that internal transcribed spacer (ITS) should be incorporated into the core barcode for seed plants. Proceedings of the National Academy of Sciences of the United States of America 108:19641–19646 DOI 10.1073/pnas.1104551108.
Mizuno T, Okuyama Y, Iwashina T. 2018. Flavonoids from Iris sanguinea var. tobataensis and chemotaxonomic and molecular phylogenetic comparisons with Iris sanguinea var. sanguinea. Bulletin of the National Museum of Nature and Science, Series B. Botany 44:135–145.
Wilson CA. 2009. Phylogenetic relationships among the recognized series in Iris section Limniris. Systematic Botany 34:277–284 DOI 10.1600/036364409788606316.
Yamamoto S, Uchida K. 2018. A generalist herbivore requires a wide array of plant species to maintain its populations. Biological Conservation 228:167–174 DOI 10.1016/j.biocon.2018.10.018.
