# Supplementary material for: Iris sanguinea is conspecific with I. sibirica (Iridaceae) according to morphology and plastid DNA sequence data
Source: PeerJ. 2020 Oct 1;8:e10088. doi: 10.7717/peerj.10088 (PMC7533061; doi:10.7717/peerj.10088)
Supplement: Supplemental Information 2 — M –mean (cm), S –standard deviation, C –coefficient of variation (%). The values in parentheses are adjusted p-values. Refer to Table 1 for character abbreviations. [file peerj-08-10088-s002.rtf]

Table S2 The results of the variance analysis of the Iris subser. Sibiricae species.
Character	PC1	PC2	PC3	p-value	Species	M±S	C	
LL	0.735	0.676	–0.039	0.000002
(0.000022)	I. sanguinea	43.9±12	27.2	
					I. sibirica	55.6±16.3	29.3	
					I. typhifolia	43.2±14.7	34.1	
LW	0.005	0.005	–0.003	>0.0000
(>0.0000)	I. sanguinea	0.4±0.2	40.1	
					I. sibirica	0.5±0.2	31	
					I. typhifolia	0.3±0.1	28.8	
SH	0.676	–0.736	–0.004	0.000003
(0.000024)	I. sanguinea	48.8±12.8	26.1	
					I. sibirica	61.2±16.5	27	
					I. typhifolia	53.1±9.7	18.3	
IS	0.006	–0.003	–0.014	0.000891
(0.008019)	I. sanguinea	1.1±0.3	26.1	
					I. sibirica	1.3±0.5	35.3	
					I. typhifolia	1.1±0.3	29.4	
NF	0.018	–0.011	–0.030	>0.0000
(>0.0000)	I. sanguinea	2.2±0.6	27.8	
					I. sibirica	2.7±1.1	39.6	
					I. typhifolia	1.9±0.6	34.8	
NC	0.011	–0.013	–0.018	>0.0000
(>0.0000)	I. sanguinea	1.4±0.6	43.5	
					I. sibirica	1.7±0.7	40	
					I. typhifolia	2.2±0.5	24.3	
CL	0.033	0.023	0.987	0.193727
(1)	I. sanguinea	7.2±4.2	59	
					I. sibirica	6.2±2.3	37.6	
					I. typhifolia	6.6±1.4	21.3	
BL	–0.003	–0.010	0.134	>0.0000
(>0.0000)	I. sanguinea	3.6±1.1	29.6	
					I. sibirica	3.2±0.7	21.9	
					I. typhifolia	4.6±0.8	16.5	
PL	–0.001	0.021	0.062	0.007416
(0.066744)	I. sanguinea	2.2±1.1	50.5	
					I. sibirica	1.7±1.4	85.5	
					I. typhifolia	2.4±1.2	50	
Notes:
M – mean (cm), S – standard deviation, C – coefficient of variation (%).
The values in parentheses are adjusted p-values.
Refer to Table 1 for character abbreviations.
