# Supplementary material for: Iris sanguinea is conspecific with I. sibirica (Iridaceae) according to morphology and plastid DNA sequence data
Source: PeerJ. 2020 Oct 1;8:e10088. doi: 10.7717/peerj.10088 (PMC7533061; doi:10.7717/peerj.10088)
Supplement: Supplemental Information 3 [file peerj-08-10088-s003.rtf]

Annex 1 Complete list of specimens examined in the morphological study.

Iris sanguinea Hormem.
CHINA. Anhui Province: Manchuria, Jalatun, [fl.], [27] Jun 1925, P.H. & J.H. Dorsett 3482 (PE01013371). Heilongjiang Province: Wutaili, Dailing, Yichun City, [fr.], 17 Jul 1956, S. Liu et al. 7219 [originally in Chinese] (PE01013369); Yichun City, [fr.], 26 Jul 1956, S. Liu et al. 7784 [originally in Chinese] (PE01013370); s.coll., [fr.], 1959, L. Zhang 268 [originally in Chinese] (PE02238594). Inner Mongolia: Mal Qihan, [fl.], 19 Jun 1986, s.coll. 68 [originally in Chinese] (PE02238601); Ali River, Yakeshi, [fl.], 11 Jul 1986, s.coll. 377 [originally in Chinese] (PE02238596); Wumenggou Manmenggou, Ulanhot, 1035 m, [fl.], 23 Jun 2009, D. Fu et al. 09062305 [originally in Chinese] (PE02238607). Jilin Province: Near Weihuling Station, 500 m, [fl.], 18 Jun 1931, U.W. Kung 1598 (E00711867! & PE01013380); Near Sanchazi, Linjiang, [fl.], 11 Jun 1950, Y. Zhou et al. 721 [originally in Chinese] (PE01013376); Linjiang City, [fl.], 12 Jun 1950, Z. Wu et al. 774 [originally in Chinese] (PE01013376); Changbai Mountain, [fl.], 1963, s.coll. 147 (PE02238603). JAPAN. Hondo: Prov. Shimotsuke, pref. Tochigi, Senjooga-hara Nikko, 1400 m, [fl.], 27 Jun 1946, M. Furuse 18174 (PE00106433); Minagawa-joonai, Tochigi-shi, pref. Tochigi, [fl.], 20 May 1991, M. Furuse 55811 (PE00106473). MONGOLIA. Dornod Aimag: Khuviyn-Bulak spring, [fl.], 18 Jun 1975, O.V. Zhurba 22 [originally in Russian] (MW0173984! & MW0173989!); 28 km north of Mungum Mort, [fl.], 3 Jul 1979, V.I. Grubov et al 200/11 [originally in Russian] (MW0173983!); Mount Khan Chandman Ula, 1100–1350 m, [fl.], 1 Jul 1987, A.L. Budantsev et al. 915 [originally in Russian] (MW0173974! & MW0173976!); 45 km southeast of Sumber, 850–900 m, [fl.], 17 Jul 1987, R.V. Kamelin et al. 1658 [originally in Russian] (MW0173972!); 35 km north of Sumber, 720–750 m, [fl.], 19 Jul 1987, Sh. Dariymaa et al. 1831 [originally in Russian] (MW0173977!); 20 km north of Sumber, 700–750 m, [fl.], 25 Jul 1987, R.V. Kamelin et al. 342 [originally in Russian] (MW0173973!); Near Mount Khan Chandman, [fl.], 22 Jul 1989, Ch. Sanchir 305 [originally in Russian] (MW0173980!); 16 km west of Bayan-Uul, [fr.], 3 Aug 1990, I.A. Gubanov 550 [originally in Russian] (MW0173981!); 45 km north of Sumber, [fr.], 4 Aug 1991, I.A. Gubanov et al. 946 [originally in Russian] (MW0173978!); Western spurs of the Greater Khingan Range, eastern Mount Khan Chandman Ula, [fr.], 8 Aug 1991, I.A. Gubanov & Sh. Dariymaa 1118 [originally in Russian] (MW0173975! & MW0173979!). Khentii Aimag: Norovlin Sum, Erendaba Range, 1200 m, [fr.], 5 Aug 1985, I.A. Gubanov 9812 [originally in Russian] (MW0173986!); 70 km south of Binder, [fr.], 14 Aug 2005, A.V. Galanin s.n. [originally in Russian] (VBGI79710!); Confluence of Barha and Onon rivers, [fr.], 6 Aug 2006, L.M. Dolgaleva s.n. [originally in Russian] (VBGI79703!); National park “Alkhanay”, [fr.], 10 Jul 2009, L.M. Dolgaleva s.n. [originally in Russian] (VBGI79704!). RUSSIA. Amur Oblast: Zavitinsky Raion, 0.5 km north-east of Kamyshenka Village, [fl.], 18 Jun 1955, I. Gubanov s.n. [originally in Russian] (MW0042368!); Mazanovsky Raion, meadow in the floodplain of Selemdzha River, [fl.], 20 Jun 1955, A. Tyrtikov s.n. [originally in Russian] (MW0042175!); Between Novovoskresenovka and Bibikovo villages, [fl.], 19 Jun 1958, G.N. Nepli s.n. [originally in Russian] (MW0042365!); Blagoveshchensky Raion, southeast of Mukhinka Village, [fl.], 7 Jun 1959, N.A. Berezina s.n. [originally in Russian] (MW0042173! & MW0042176!); Zeysky Raion, near Sosnovy Bor, [fl.], 2 Jun 1978, M.S. Ignatov s.n. [originally in Russian] (MW0042364!); Zeysky Raion, Zeysky State Nature Reserve, along the Zolotogorsk highway 55 km from Zeya, [fl.], 16 Jun 1978, I.A. Gubanov s.n. [originally in Russian] (MW0042362! & MW0042363!); Arkharinsky Raion, 45 km south of Uril Village, near Peresheyechnoye Lake, [fl.], 18 Jun 1985, V.V. Yakubov & G.E. Fedoseev s.n. (MW0042171!); Near Blagoveshchensk, [fr.], 16 Jun 2000, G.F. Darman s.n. [originally in Russian] (VBGI79707! & VBGI79708!); Khingan Nature Reserve, [fl.], 9 Jun 2016, S.G. Kudrin s.n. [originally in Russian] (VBGI35982!); 1.5 km below the confluence of Lucha and Zeya rivers, 900 m, [fr.], 15 Aug 2018, T.V. Stupnikova & I.G. Borisova s.n. [originally in Russian] (ABGI64768). Buryatia: Near Verkhneudinsk [Ulan-Ude], [fl.], 5 Jun 1913, G. Poplavskaya et al. 485 [originally in Russian] (MW0042190!); Kudarinsky [Kabansky] Raion, the Chikoy River valley, [fl.], 26 Jun 1954, s.coll. s.n. [originally in Russian] (NSK0068609!); Khorinsky Raion, Bayan-Gol Village, [fl.], 2 Jul 1954, Eiges & Belyaeva s.n. [originally in Russian] (MW0042183!); 40 km from Vitim to Romanovka Village, [fl.], 16 Jul 1961, I. Sukacheva s.n. [originally in Russian] (MW0042191!); Bauntovsky Raion, near Rossoshino Village, [fl.], 10 Jul 1964, s coll. s.n. [originally in Russian] (MW0042185!); Kharatsay Village, the Ulyatuy River valley, [fl.], 10 Jun 1965, G. Peshkova & Skudenkova 678 [originally in Russian] (NSK0068605!); Podlopatki Village, [fr.], 14 Jul 1965, G. Peshkova & Tarasova 2183 [originally in Russian] (NSK0068608!); Khorinsk Village, the Uda River valley, [fr.], 8 Aug 1965, G. Peshkova & Skudenkova 2863 [originally in Russian] (NSK0068095!); Mukhorshibirsky Raion, 2.5 km south of Podlopatki Village, [fl.], 7 Jun 2012, L. Abramova et al. s.n. [originally in Russian] (MW0156287!). Irkutsk Oblast: Lake Baikal, near Yelantsy Village, [fl.], 22 Jun 1956, A.G. Telpukhovskaya s.n. [originally in Russian] (NSK0068096!); Olkhonsky Raion, Chernorud Village, [fl.], 27 Jun 2006, S.G. Kazanovskiy 294 [originally in Russian] (NS0033571!). Primorsky Krai: [Lazovsky Raion], Sikhote-Alin mountain range, 3 km north of Chernoruchye Village, [fl.], 15 Jun 1945, P. Zhudov 1151 [originally in Russian] (MW0042366!); Shkotovsky Raion, Kangauz [Anisimovka Villag], [fl.], 25 Jun 1969, Zozulyuk s.n. [originally in Russian] (VBGI79691!); Khankaysky Raion, fish farming Khankaysky, [fl.], 8 Jun 1970, L. Boiko s.n. [originally in Russian] (VBGI79676!); Oktyabrsky Raion, near Galyonki Village, [fl.], 7 Jun 1973, V.P. Verholat s.n. [originally in Russian] (VBGI79673! & VBGI79674!); Shkotovsky Raion, Anisimovka Village, [fl.], 11 Jun 1975, s.coll. s.n. [originally in Russian] (VBGI79572!); Lazovsky Raion, Kiyevka Village, [fl.], 29 Jun 1977, C. Minina s.n. [originally in Russian] (VBGI79683!); Pozharsky Raion, [fl.], 3 Jun 1982, I.V. Shibneva s.n. [originally in Russian] (VBGI79684!); Khankaysky Raion, near Dvoryanka Village, [fl.], 19 Jun 2005, A.V. Galanin s.n. [originally in Russian] (VBGI79670!); In the vicinity of Artemovo Village, left bank of the Serebryanka River, [fl.], 15 Jun 2014, E.A. Pimenova s.n. [originally in Russian] (VBGI35384!, the epitype of I. sanguinea). Sakha Republic: Ust-Aldansky Raion, [fl.], 1 Sep 1951, V. Kuvaev 119 [originally in Russian] (MW0042179!). Zabaykalsky Krai: Yamarovka Village, [fl.], 20 Jun 1892, P. Mikhno 69 [originally in Russian] (NSK0068583!); Byrkinsky [Priargunsky] Raion, between Novotsuruhaitui and Sorgol, [fl.], 16 Jun 1931, M.I. Nazarov 13.617 [originally in Russian] (MW0042187!); Near Borzya [fl.], 19 Jun 1953, G.A. Peshkova & B.M. Khaptagayev s.n. [originally in Russian] (NSK0068580!); Nerchinsky Zavod Village, [fl.], 29 Jun 1953, G.A. Peshkova & B.M. Khaptagayev s.n. [originally in Russian] (NSK0068586!); Aginsky Raion, south of Datsan, [fl.], 17 Jun 1956, Blinova & Mikhalkina 13/4 (MW0042195!); Kalgansky Raion, Srednyaya Borzya Village, [fl.], 5 Jul 1960, Nomokonov & Zarubin s.n. [originally in Russian] (NSK0068591!); Ononsky Raion, Novyy Durulguy Village, [fr.], 19 Jul 1960, Peshkova & Karbina 119 [originally in Russian] (NSK0068590!); Olovyanninsky Raion, Nizhniy Sharanay Village, [fl.], 28 Jun 1961, Peshkova & Karbina 245 [originally in Russian] (NSK0068581!); Baley, Sredniy Golgotay Stream [fr.], 8 Aug 1963, G. Peshkova & Martynova s.n. [originally in Russian] (NSK0068602!); Argunsk Village, [fr.], 6 Sep 1963, Peshkova & Martynova s.n. [originally in Russian] (NSK0068593!); Aginskoye, [fl.], 12 Jun 1964, G. Peshkova & L. Turova s.n. [originally in Russian] (NSK0068596!); Nerchinsk, Olinsk Village, [fl.], 18 Jun 1964, G. Peshkova & L. Turova s.n. [originally in Russian] (NSK0068604!); Kyra Village, [fr.], 30 Jul 1964, G. Peshkova & L. Ovchinnikova s.n. [originally in Russian] (NSK0068594!); Mogochinsky Raion, Razdol'noye, [fl.], 23 Jun 1966, Blagodatskikh 125 [originally in Russian] (MW0042188!); Mogochinsky Raion, Teterkin Klyuch Village, [fr.], 24 Jul 1967, Podugolnikova 126 [originally in Russian] (MW0042371!); Mogoytuysky Raion, 8 km north of Dogoy Village, [fl.], 16 Jun 1972, V. Kuvaev et al. 187-3 [originally in Russian] (MW0042370!); Verkhniy Bukun, [fr.], 19 Jul 2001, A.V. Galanin s.n. [originally in Russian] (VBGI81549!); 2 km south of Verkhniy Ul'khun Village, [fr.], 31 Jul 2001, A.V. Galanin s.n. [originally in Russian] (VBGI81545!); Kyrinsky Raion, 20 km south of Yuzh. Argaley, 662 m, [fl.], 15 Jun 2004, V.M. Doronkin s.n. [originally in Russian] (NSK0008006!); Kyrinsky Raion, Sokhondo Nature Reserve, [fl.], 26 Jun 2004, V.M. Doronkin s.n. [originally in Russian] (NSK0005997!); 2 km from Uryupino Village, [fr.], 20 Jul 2004, A.V. Galanin s.n. [originally in Russian] (VBGI79702!); Kyra River, [fr.], 21 Jul 2004, L.M. Dolgaleva s.n. [originally in Russian] (VBGI81550!); Regional reserve “Gornaya step”, [fr.], 22 Jul 2005, E.N. Roenko s.n. [originally in Russian] (VBGI79693! & VBGI79695!); Regional reserve “Gornaya step”, [fl.], 6 Jun 2010, E.N. Roenko s.n. [originally in Russian] (VBGI81548!); Aginsky Raion, near Tsokto-Khangil Village, [fr.], 15 Jul 2011, N.S. Gamova & S.V. Dudov 11-0345 [originally in Russian] (MW0042180!).

Iris sibirica L.
AUSTRIA: Niederösterreich, Pischelsdorfer Wiesen, 170 m, [fr.], 22 Aug 2013, F. Tod et al. FT20130822-8 (WU0075061 & WU0075062); Standort, Niederosterr, Sumpfwiesen bei Laxenburg, [fl.], 26 May 1904, L. Keller s.n. (PI-GUAD No. 023800). BELARUS: Loyew Raion, [fl.], 17 Jun 1935, Dvorakovsky s.n. [originally in Russian] (MW0294396); Maladzyechna Voblast, the Yelnya swamps, [fl.], 3 Jul 1958, L. Denisova s.n. [originally in Russian] (MW0294397!). BULGARIA: In graminosis declivium m. Vitoš, [fl.], 1887, J. Velenovský s.n. (PRC451281). GEORGIA: Region Borjomi, Bakuriani, Bakuriani Botanical Garden, [fr.], 8 Aug 1998, M. Merello et al. (E00283854!). ITALY: Venetia, Prov. di Treviso, Vittorio, 100 m, [fl.], 18 May 1904, A. Fiori et al. s.n. (PI-GUAD No. 023799). KAZAKHSTAN: Kustanai Uyezd, [fr.], 18 Jul 1925, F. Usanova s.n. [originally in Russian] (MW0816529!). LATVIA: Liepajas Raion, 5 km north-east of Rucava, [fr.], 27 Jul 1989, N. Shvedchikova s.n. [originally in Russian] (MW0294379!). MONGOLIA: Selenge Aimag, Eroo Sum, tract Honin Nug, [fl.], 16 Jun 2012, D.N. Shaulo 14 [originally in Russian] (NS0030801!). RUSSIA: Altai Republic, Shebalinsky Raion, near Chemal, [fr.], 28 Jul 1984, M. Lomonosova 2528 [originally in Russian] (NSK0068568!); Arkhangelsk Oblast, the Solovetsky Islands, Bolshaya Muksalma Island, [fl.], 17 Jul 2002, V. Novikov et al. s.n. [originally in Russian] (MW0564809!); Bashkortostan, Baymaksky Raion, 7–8 km west of Baymak, [fl.], 13 Jun 1958, P.P. Zhudova & L. Dunaeva s.n. [originally in Russian] (MW0294579!); Bryansk Oblast, Trubchevsk Village, [fl.], 19 Jun 1926, V.A. Arseniev s.n. [originally in Russian] (MW0294392!); Bryansk Governorate, Trubchensk Village, [fl.], 20 Jun 1926, Snyatinovskaya s.n. [originally in Russian] (MW0294391!); [Eastern Administrative Okrug], Ukhtomsky Raion, between Korsievo and Torbeevo villages, [fl.], 7 Jun 1946, B. Kulkov 41 [originally in Russian] (MHA0032146!); Ivanovo Oblast, Savinsky Raion, north east of Kniginkino Village, [fl.], 19 Jun 1957, N.V. Rau s.n. [originally in Russian] (MW0294404!); Kaluga Oblast, Sukhinichsky Raion, between Frolovo and Zherdevo villages, [fr.], 14 Aug 1972, I.A. Gubanov s.n. [originally in Russian] (MW0294406!); [Kaluga Oblast], near Kaluga, [fl.], 11 Jun 1998, S. Mayorov et al. s.n. [originally in Russian] (MW0294417!); Karachay-Cherkess Republic, Karachayevsky Raion, the Uchkulan River gorge, 15 km above the Verkhniy Uchkulan Village, 1630 m, [fl.], 5 Aug 2006, A.S. Zernov & I.I. Shidakov 5349 [originally in Russian] (MW0657663!); Kostroma Oblast, Manturovsky Raion, Ugory Village, [fl.], 11 Jul 1987, s.coll. s.n. [originally in Russian] (MW0294507!); [Krasnodar Krai], Caucasian State Nature Reserve, [fl.], 3 Aug 1960, Khudyakova s.n. [originally in Russian] (MW0657662!); Kurgan Oblast, Pritobolny Raion, near Ukrainets Village, [fl.], 18 Jun 1984, V. Doronkin 7 [originally in Russian] (NSK0068578!); Kurgan Oblast, distr. Gladjanskoe, prope pag. Zverinogolovsk, [fl.], 19 Jun 1984, V. Doronkin s.n. Exs. No. 6645 (MW0042061! & NS0033547!); Mari El Republic, Medvedevsky Raion, 4 km east of Kuzhinskiy Konoplyanik Village, Mazarskoye Lake, [fl.], 12 Jun 1996, V.N. Tikhomirov et al. s.n. [originally in Russian] (MW0294554!); Mordovia Republic, Tengushevsky Raion, Standrovo Village, [fl.], 13 Jul 1979, T. Silaeva s.n. [originally in Russian] (MW0294551!); Mordovia Republic, Tengushevsky Raion, 2 km southeast of Shiromasovo Village, Chernoye Lake, [fr.], 3 Aug 1999, T. Kramina et al. [originally in Russian] (MW0294555!); Moscow Oblast, Serpukhov Raion, the Avangard station, [fl.], s.d., V.I. Sobolevski s.n. (MHA0032152! & MHA0032153!); Moscow Oblast, in paludibus silvosis inter p. Ruposowo at Czerkizowo distr. Mosqua, [fl.], 6 Jun 1898, D. Syrejsczikow s.n. Exs. No. 236 (MW0294438-1! & MHA0032144!); Moscow Oblast, Pushkino, [fl.], 11 Jun 1907, D.P. Syreyshchikov 1455 [originally in Russian] (MW0294453!); Moscow Oblast, Serpukhovsky Raion, Luzhki Village, [fl.], 1 Jun 1946, P. Smirnov s.n. [originally in Russian] (MW0294461!); Moscow Oblast, Serpukhovsky Raion, Luzhki Village, [fl.], 12 Jun 1948, T. Gordeeva s.n. [originally in Russian] (MW0294477!); Moscow Oblast, Serpukhov Raion, near Luzhki Village, [fr.], 26 Jul 1953, V.I. Sobolevski s.n. [originally in Russian] (MHA0032140! & MHA0032142!); Moscow Oblast, Serpukhov Raion, near Luzhki Village, [fl.], 31 May 1967, E.I. Kurchenko s.n. (MHA0032138!); Moscow Oblast, Mytishchinsky Raion, near Veshki Village, [fl.], 15 Jun 2016, K. Yu. Teplov s.n. [originally in Russian] (MHA0020928!); Nizhny Novgorod Governorate, Sergachsky Uyezd, between Pozharki and Yendovishchi villages, [fl.], 8 Jun 1926, P. Smirnov s.n. [originally in Russian] (MW0294536!); [Nizhny Novgorod Oblast], Krasnobakovsky Uyezd, Zavody Village, [fl.], 26 Jun 1928, D. Averkiev & L. Gorokhova s.n. [originally in Russian] (MW0294550!); Nizhny Novgorod Oblast, Vodovatovo Village, [fl.], 13 Jun 1929, Snyatinovskaya s.n. [originally in Russian] (MW0294539!); North Ossetia-Alania Republic, North Ossetia Nature Reserve, south slope of Tsey Gorge, 2500, [fl.], 16 Jul 1976, A.M. Amirkhanov s.n. [originally in Russian] (MW0657672!); North Ossetia-Alania Republic, Bad Gorge, [fl.], 28 Jun 1977, A.M. Amirkhanov s.n. [originally in Russian] (MW0657673!); North Ossetia-Alania Republic, south slope of Tsey Gorge, [fl.], 28 Jun 1977, N. Shvedchikova s.n. [originally in Russian] (MW0657671! & MW0657676); [North Ossetia-Alania Republic], Caucasus Magnus, Ossetia Borealis, distr. Vladikavkaz, iugum montimum Skalystyj khrebet, vicinitas pagi Fiogdon, 2000 m, [fr.], Aug 1987, J. Èuba s.n. (E00332752!); Novosibirsk Oblast, Chanovsky Raion, [fl.], 11 Jun 1949, T. Vagina s.n. [originally in Russian] (NS0033551!); Novosibirsk Oblast, near Kuybyshev, [fl.], 21 Jun 1949, T. Vagina s.n. [originally in Russian] (NS0033553!); Novosibirsk Oblast, Chanovsky Raion, [fl.], 31 May 1954, T. Vagina s.n. [originally in Russian] (NSK0068570!); Novosibirsk Oblast, Tatarsky Raion, Kazachiy Mys Village, [fr.], 3 Aug 1954, T. Vagina s.n. [originally in Russian] (NS0033550!); Novosibirsk Oblast, Kolyvansky Raion, near Chaus Village, [fl.], 12 Jun 1956, E. Penkovskaya & A. Buturlina s.n. [originally in Russian] (NS0033549!); Novosibirsk Oblast, Ordynsky Raion, [fl.], 20 Jun 1957, E. Penkovskaya & A. Buturlina s.n. [originally in Russian] (NS0033548!); Omsk Oblast, near Elizavetinka Village, [fr.], 20 Jul 1984, A. Krasnikov & S. Bubnova 2233 [originally in Russian] (NSK0068571!); Omsk Oblast, Novovarshavsky Raion, Novovarshavka Settlement, [fl.], 15 Jun 2007, A. Batyakova s.n. [originally in Russian] (NSK0005456!); Pskov Oblast, near Mikhaylovskoye, [fr.], 13 Aug 1999, Yu. Alekseev s.n. [originally in Russian] (MW0294376!); Ryazan Oblast, Spassky Raion, kordon Tyshlovo, [fl.], 12 Jun 1966, V. Tikhomirov & E. Aksenov s.n. [originally in Russian] (MW0294426!); Ryazan Oblast, Spassky Raion, between Gorodkovichi and Izhevskoye villages, [fl.], 2 Jun 1968, V. Tikhomirov & S. Alexander s.n. (MW0294420!); Ryazan Oblast, Spassky Raion, kordon Tyshlovo, [fl.], 6 Jun 1968, V. Tikhomirov et al. s.n. [originally in Russian] (MW0294425!); Ryazan Oblast, Spassky Raion, Oka Nature Reserve, [fl.], 24 Jun 1974, V. Tikhomirov & T. Dyachenko 9017 [originally in Russian] (MW0294427!); Samara Oblast, Saratov Reservoir, [fl.], 16 Jun 1973, V. Ekzertsev & V. Artemenko s.n. [originally in Russian] (MW0294563!); Smolensk Oblast, Yartsevsky Raion, Mikheykovo Village, [fl.], 1 Jul 1957, Multanovskaya s.n. [originally in Russian] (MW0294386!); Smolensk Oblast, Ugransky Raion, 16 km west of Vskhody Village, [fl.], 14 Jul 1998, A.A. Shmytov s.n. [originally in Russian] (MW0294384!); Smolensk Oblast, Demidovsky Raion, 7–8 km west of Baklanovo Village, [fl.], 14 Jun 2000, Ya.V. Kosenko & N.M. Reshetnikova s.n. [originally in Russian] (MW0294385!); Smolensk Oblast, Demidovsky Raion, Shchuch'ye Lake, 1 km north of Mitino Village, [fl.], 15 Jun 2001, N.M. Reshetnikova s.n. [originally in Russian] (MW0294394!); [Stavropol Krai], 43 km Kislovodsk – Karachaevsk highway, [fl.], 6 Jul 1976, E.E. Gogina s.n. [originally in Russian] (MW0657669!); Tomsk Governorate, Kainsky Uyezd, Borodina Village [Kutashevo], [fl.], 11 Jun 1912, B.N. Klopotov s.n. [originally in Russian] (NSK0068569!); Tver Oblast, Zapadnodvinsky Raion, near Barondo Village, [fr.], 19 Aug 1994, A. Notov et al. s.n. (MW0294375!); Tyumen Oblast, Savino Village, [fl.], 12 Jun 1908, S. Mamaev 19 [originally in Russian] (NSK0068573!); Tyumen Oblast, Isetsky Raion, between Salabaevo and Borovlyanka villages, [fl.], 8 Jun 1961, N.A. Yudkina s.n. [originally in Russian] (NSK0068574!); Tyumen Oblast, Zavodoukovskiy Raion, Komissarovsky forestry, [fl.], 7 Jul 1979, M.S. Ignatov s.n. [originally in Russian] (MW0042060!); Tyumen Oblast, Abatsky Raion, near Starovyatkina Village, [fr.], 16 Jul 1984, S. Bubnova 2015 [originally in Russian] (NSK0068575!); Ural Oblast, Nadezhdinsky Raio, near Morozovo Village, 32 km southeast of Nadezhdinsk, [fr.], 30 Aug 1933, N. Sokolova s.n. [originally in Russian] (MW0294565!); Vladimir Oblast, Petushinsky Raion, 2.5 km east of Bogdarnia Village, [fl.], s.d., A. Savena s.n. [originally in Russian] (MW0294419!); Vladimir Oblast, Sudogodsky Raion, Spas-Kupalishche, [fl.], 18 Jun 1969, V. Tikhomirov et al. s.n. [originally in Russian] (MW0294431!); Vladimir Oblast, Kameshkovsky Raion, near Rusino Village, [fl.], 12 Jun 1971, V. Tikhomirov et al. 5048 [originally in Russian] (MW0294430!); Vladimir Oblast, Petushinsky Raion, [fl.], 19 Jun 2006, M.G. Berezkina s.n. [originally in Russian] (MW0294418!); Yaroslavl Oblast, Breytovsky Raion, Kordon Yana, [fl.], 23 Jun 1977, L. Lisitsyna s.n. [originally in Russian] (MW0294512!). TURKEY: Prov. Kars, Yalnizçam dað between Yalnizçam & Ardanuç, above the castle, 2250 m, [fl.], 16 Jun 1957, Davis & Hedge D.29664 (E00332753!); Prov. Kars, Ziyaret dað (Yalnizçam Daðlari), between Ardahan & Artvin, 2250 m, [fl.], 29 Jun 1957, Davis & Hedge D.30283 (E00332751!); Prov. Kars, SW slope of Kisir dað, 2500 m, [fl.], 3 Jul 1957, Davis & Hedge D.30561 (E00332755!); Prov. Kars, Yagmurlu dað between Sarikamiþ & Karaurgan, 2200 m, [fl.], 7 Jul 1957, Davis & Hedge D.30704 (E00332754!). UKRAINE: Zakarpattia Oblast, Khust Raion, Kireshi Village, [fl.], 30 May 1984, A.P. Efremov s.n. [originally in Russian] (MW0294584!).

Iris typhifolia Kitag.
CHINA: s.loc., [fl.], s.d., s.coll. s.n. (NENU00018298!); s.loc., [fl.], 14 Jun 1950, M. Noda et al. 327 [originally in Chinese] (PE01013772); s.loc., [fl.], 8 Jun 1960, J. Zhao s.n. [originally in Chinese] (NENU!); s.loc., [fl.], 11 Mar 1974, Y. Zhao 17 [originally in Chinese] (NENU00018245!); Hebei Province, [fl.], 20 Jun 1927, [É. Licent] 8083 (PE01013778); Hebei Province, [fl.], 21 Jun 1927, [É. Licent] 8107 (PE01013779); Heilongjiang Province, [fr.], 29 May 1930, O. Gosei s.n. [originally in Japanese] (IFP15405013x0015); Heilongjiang Province, Harbin City, [fl.], 8 Jun 1949, s.coll. 197 [originally in Chinese] (IFP15405013x0009); Heilongjiang Province, Tahe County, 300 m, [fr.], 20 Jul 1950, D. Zhao 176 [originally in Chinese] (IFP15405013x0013); Heilongjiang Province, Huma County, 360 m, [fr.], 5 Aug 1950, L. Han 682 [originally in Chinese] (IFP15405013x0010); Heilongjiang Province, [fr.], 22 Sep 1951, D. Zhao 990 [originally in Chinese] (PE01013773 & IFP15405013x0007); Heilongjiang Province, Heihe City, [fl.], 26 Jun 1957, G. Cui 1039 [originally in Chinese] (IFP15405013x0011); Heilongjiang Province, Heihe City, [fr.], 7 Jul 1957, G. Cui 1163 [originally in Chinese] (IFP15405013x0014); [Heilongjiang Province], Shuangfeng, [fl.], Jun 1960, s.coll. 105 [originally in Chinese] (NENU00018239! & NENU00018244!); [Heilongjiang Province], Anda City, Sifang Mountain, [fr.], 18 Jul 1960, s.coll. 061 [originally in Chinese] (NENU00018309!); [Heilongjiang Province], Anda City, Lamadian Town, [fl.], 19 Jul 1960, G. Zhang 203 [originally in Chinese] (NENU00018310!); [Heilongjiang Province], Qinggang County, New Village, [fr.], 22 Jul 1960, s.coll. 334 [originally in Chinese] (NENU00018242! & NENU00018305!); [Heilongjiang Province], Qinggang County, [fl.], 26 Jul 1960, C. Liu 180 [originally in Chinese] (NENU00018241! & NENU00018304!); Heilongjiang Province, Taikang County, [fr.], 29 Jul 1960, s.coll. 135 [originally in Chinese] (NENU!); Inner Mongolia, Yakeshi, [fl.], 7 Jul 1949, s.coll. 1293 [originally in Chinese] (IFP15405013x0021); Inner Mongolia, Aershan City, [fl.], 14 Jun 1950, Y. Zhang 327 [originally in Chinese] (IFP15405013x0020); [Inner Mongolia], Yihuta Town, Tala grassland, [fr.], 25 Jul 1955, Takeuchi s.n. [originally in Chinese] (NENU00018247!); Inner Mongolia, Hailar City, 590 m, [fl.], 10 Jun 1951, Z. Wang et al. 633 [originally in Chinese] (KUN0360611 & PE01013771); Inner Mongolia, [fl.], 29 Jun 1984, Medicine Team 2272 [originally in Chinese] (IFP15405013x0023); Jiangsu Province, [fl.], 13 May 1926, C.L. Tso 447 (PE01013780); [Jilin Province], Bei Ling, [fl.], 4 Jun, s.coll. s.n. [originally in Chinese] (NENU!); Jilin Province, Shuangliao County, [fl.], 4 Jun 1950, Y. Zhang 24 [originally in Chinese] (IFP15405013x0004); [Jilin Province], Taonan City, Heishui Town, [fl.], Jul 1957, L. Zhao s.n. [originally in Chinese] (NENU00018246! & NENU00018297!); [Jilin Province], Da'an City, Yueliangpao Town, [fl.], 26 May 1959, The First Team of Baicheng 1018 [originally in Chinese] (NENU00018302! & NENU00018307!); [Jilin Province], Yueliangpao Town, [fl.], 26 May 1959, The Second Team of Baicheng 2018 [originally in Chinese] (NENU00018249!); [Jilin Province], Baicheng City, Yueliangpao Town, [fl.], 25 May 1959, The Third Team of Baicheng 3018 [originally in Chinese] (NENU00018300!; NENU00018301! & NENU00018308! – “26 May 1959”); [Jilin Province], Tongyu County, [fl.], 21 Jun 1959, The First Team of Baicheng 5039 [originally in Chinese] (NENU00018243!); [Jilin Province], Tongyu County, Cattle farm, [fl.], 10 Jun 1960, J. Ye 694 [originally in Chinese] (NENU! & NENU00018303!); Jilin Province, Tongyu County, [fl., fr.], 16 Jun 1960, J. Ye 69 [originally in Chinese] (PE01013774–PE01013776); [Jilin Province], Shuangliao Bao City, Baoshitu Village, [fl.], 8 Jun 1963, Yang & D. Jing 045 [originally in Chinese] (NENU!); [Liaoning Province?], BeiLing, [fl.], s.d., s.coll. s.n. [originally in Chinese] (NENU00018248!).
